# The influence of leaf anatomy on the internal light environment and photosynthetic electron transport rate: exploration with a new leaf ray tracing model

**DOI:** 10.1093/jxb/erw359

**Published:** 2016-10-04

**Authors:** Yi Xiao, Danny Tholen, Xin-Guang Zhu

**Affiliations:** ^1^CAS Key Laboratory of Computational Biology and State Key Laboratory of Hybrid Rice, CAS-MPG Partner Institute for Computational Biology, Shanghai Institutes for Biological Sciences, Chinese Academy of Sciences, Shanghai 200031, China; ^2^University of Chinese Academy of Sciences, 19A Yuquan Road, Beijing 100049, China; ^3^Institute of Botany, Department of Integrative Biology, University of Natural Resources and Applied Life Sciences, BOKU Vienna, Gregor Mendel-Str. 33, A-1180 Vienna, Austria

**Keywords:** Convexity, detour effect, focusing effect, leaf anatomy, photoinhibition, ray tracing model, sieve effect.

## Abstract

Leaf photosynthesis is determined by biochemical properties and anatomical features. Here we developed a three-dimensional leaf model that can be used to evaluate the internal light environment of a leaf and its implications for whole-leaf electron transport rates (*J*). This model includes (i) the basic components of a leaf, such as the epidermis, palisade and spongy tissues, as well as the physical dimensions and arrangements of cell walls, vacuoles and chloroplasts; and (ii) an efficient forward ray-tracing algorithm, predicting the internal light environment for light of wavelengths between 400 and 2500nm. We studied the influence of leaf anatomy and ambient light on internal light conditions and *J*. The results show that (i) different chloroplasts can experience drastically different light conditions, even when they are located at the same distance from the leaf surface; (ii) bundle sheath extensions, which are strips of parenchyma, collenchyma or sclerenchyma cells connecting the vascular bundles with the epidermis, can influence photosynthetic light-use efficiency of leaves; and (iii) chloroplast positioning can also influence the light-use efficiency of leaves. Mechanisms underlying leaf internal light heterogeneity and implications of the heterogeneity for photoprotection and for the convexity of the light response curves are discussed.

## Highlight

A new model of the dicot leaf shows that different chloroplasts can experience drastically different light conditions and that bundle sheath extensions and chloroplast positioning influence photosynthetic light use efficiency.

## Introduction

The classic steady-state biochemical model of C_3_ photosynthesis (Farquhar *et al.*, 1980; von Caemmerer, 2013) and also some dynamic models (Pearcy *et al.*, 1997; Poolman *et al.*, 2000; Laisk *et al.*, 2006; Zhu *et al.*, 2013) implicitly assume no heterogeneity of light environment inside the leaf. However, there is convincing evidence indicating that the leaf internal light environment is heterogeneous and this heterogeneity has consequences for photosynthetic efficiency (e.g. Vogelmann *et al.*, 1989; Bornman *et al.*, 1991; Cui *et al.*, 1991; Vogelmann & Martin, 1993; Takahashi *et al.*, 1994; Vogelmann & Han, 2000; Vogelmann & Evans, 2002; Tholen *et al.*, 2012). The heterogeneity of light inside the leaf largely determines the proportion of cells or chloroplasts in which photosynthesis is limited by Rubisco *versus* those where photosynthesis is limited by RuBP regeneration, and this subsequently determines the CO_2_ uptake rate of a whole leaf. The importance of leaf anatomy for leaf photosynthetic physiology is reflected by the fact that leaves that develop under different light levels also show different anatomical features (Boardman, 1977; Terashima *et al.*, 2001; Oguchi *et al.*, 2003; Terashima *et al.*, 2006).

Many empirical observations have been made regarding the influence of leaf anatomical features on the internal light environment. Lens-shaped epidermal cells can potentially focus light within the upper layers of a leaf when the incident light is perpendicular to the leaf surface, resulting in a high photon flux density (PFD) in these regions (Haberlandt, 1914; Bone *et al.*, 1985; Lee, 1986; Poulson & Vogelmann, 1990; Vogelmann *et al.*, 1996; Brodersen and Vogelmann, 2007). Columnar palisade cells minimize light scattering and may enable light to penetrate deeper into the leaf (Terashima & Saeki, 1983; Knapp *et al.*, 1988; Vogelmann *et al.*, 1988; Cui *et al.*, 1991; Vogelmann & Martin, 1993). Bundle sheath extensions (BSEs), which are strips of tightly connected chlorophyll-free ground tissue (parenchyma, collenchyma or sclerenchyma) that connect the vascular bundles with the epidermis in some leaves, may enhance the light environment within deep mesophyll regions (Karabourniotis *et al.*, 2000; Nikolopoulos *et al.*, 2002) in addition to proposed functions in mechanical support and water conduction (Wylie, 1943). Some intracellular anatomical features, such as chloroplast number, chloroplast size and arrangement, can also influence the leaf internal light environment. Specifically, chloroplasts can be located either in a profile position, i.e. arranged along the side of mesophyll cells, parallel to the direction of the light, or in the face position, i.e. along the upper or lower surface of cells, perpendicular to the light, under high light and low light, respectively (Senn, 1908; Zurzycki, 1955; Zurzycki, 1961; Davis *et al.*, 2011; Davis and Hangarter, 2012). Such changes in the position of chloroplasts can rapidly alter the absorption of light at a leaf level without requiring changes in the structure of the leaf tissue and are related to avoidance of photodamage (Kasahara *et al.*, 2002).

The influence of leaf anatomical features on the leaf internal light environment relies on the external light environment, such as incident light levels and the direction and wavelength of the incident light. Leaf absorptance is 2–3% lower when the leaf is illuminated with diffuse light compared with direct light, while leaf reflectance increases and transmittance decreases (Brodersen and Vogelmann, 2007). Palisade cells can facilitate deeper penetration of light into the mesophyll. However, the magnitude of this effect decreases under diffuse light compared with direct light (Vogelmann & Martin, 1993). In addition to the direction, the wavelength of the incident light is another important factor affecting the impact of anatomy on the internal light environment. Many components of the leaf can absorb light of different wavelengths, e.g. leaf pigments mainly absorb light in the visible wavelength region (400–700nm), while water absorbs much light in the infrared region (1400–2500nm) (Jacquemoud and Baret, 1990). Because leaf pigments have different absorption coefficients for light of different wavelengths, the spectrum of light inside a leaf gradually shifts with depth into a leaf. For example, green light is absorbed less by chlorophyll compared with red and blue light, which results in more green light penetrating to lower layers of a leaf, i.e. the gradient of green light is less steep compared with those for blue and red light (Terashima *et al.*, 2009).

Much of the above-discussed understanding of the influence of leaf anatomy and external light conditions on the light environment inside a leaf is obtained through experimental methods. A number of highly specialized instruments have been designed for this purpose (Vogelmann *et al.*, 1991; Nishio *et al.*, 1993; Takahashi *et al.*, 1994; Vogelmann & Han, 2000; Oguchi *et al.*, 2011). Although these methods help us gain substantial knowledge of the influence of leaf anatomy on the leaf internal light condition, the great variation and complexity of leaf anatomy make it difficult to use these approaches to dissect precisely the contribution of each individual anatomical feature on the light environment inside a leaf.

Mathematical modeling has long been recognized as an effective tool to study leaf optical properties. In some earlier modeling efforts, leaf anatomy was simplified as a homogeneous layer or multiple layers, and light propagation was modeled as several fluxes that were classified as either upward or downward and either direct or diffuse (Baranoski and Rokne, 2004; Ustin, 2004; Féret *et al.*, 2008). Among those various models, the ray-tracing method (Govaerts *et al.*, 1996; Ustin *et al.*, 2001) is advantageous in that it can include not only a three-dimensional representation of leaf anatomy but also a detailed description of incident light properties such as intensity, wavelength and incident angle. Recently, the anatomy of a tomato leaf has been combined with a ray tracing algorithm to predict the optical properties (Watté *et al.*, 2015) and further to simulate the photosynthetic properties (Ho *et al.*, 2016), which enables exploration of the functional implications of anatomical variations in leaves.

Here we developed a programmable 3D ray tracing model of a leaf, which can predict not only the light environment inside a leaf, but also whole leaf optical properties, such as the leaf absorptance, transmittance and reflectance. As a first application of the model, we examined the impacts of varying several anatomical features, such as bundle sheath extensions, chloroplast arrangement and lens-shaped epidermal cells, on the internal light environment. We also examined the effect of differences in the optical properties of the light (wavelength, diffuse or collimated) on the gradient in the leaf. We further studied the influence of anatomy on the shape of the light response curve of electron transport rate (*J*). The *J* of a leaf is the summation of the electron transport rate of all chloroplasts in a leaf. Based on available experimental data (Terashima and Saeki, 1985; Ögren and Evans, 1993), it is assumed that at the chloroplast level, there is no gradual and smooth transition between the light-limited and capacity-limited electron transport rates. Thus we assumed that the electron transport rate at the chloroplast level can be approximated as the minimum of these two rates. Given the different light levels absorbed by individual chloroplasts inside a leaf, here we analysed the anatomical influence on the gradual transition in the light response curve at the leaf level. Furthermore, because this new model can simulate the light absorptance of individual chloroplasts, we further characterized the ambient incident light levels needed to saturate individual chloroplasts inside the leaf.

## Materials and methods

### Model geometry construction

**Fig. 1.**
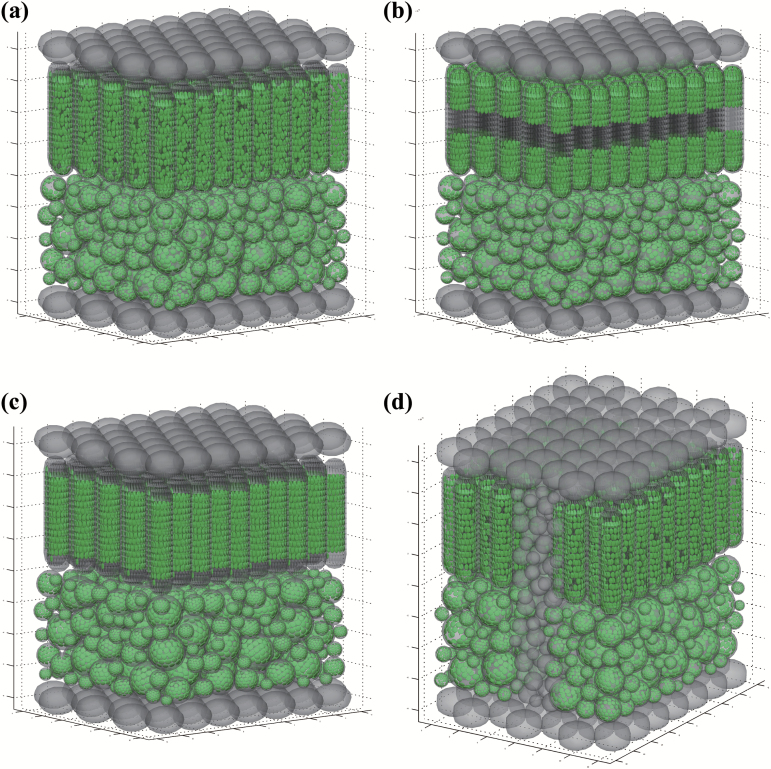
(a) The geometry of a typical dicot leaf constructed based on parameters given in Table 1. The size of modeled leaf section is about 150 µm×150 µm×180 µm. (b) Model geometry of a leaf with chloroplasts in face position. (c) Model geometry of a leaf with chloroplasts in profile position. (d) Model geometry of a leaf where bundle-sheath extensions (BSEs) making up 20% of the total leaf volume.

Figure 1a shows a three-dimensional (3D) schematic representation of a typical dicot leaf. We used the same strategy and parameters for the reconstruction of cellular shape and size as outlined in Govaerts *et al.* (1996). In summary, the geometry consisted of three leaf tissues: epidermis, palisade parenchyma and spongy parenchyma. The cells of the upper and lower epidermis were simplified as compact ellipsoid cells (Fig. 1). A palisade cell was represented by a cylinder with two hemispherical caps. Spongy cells were represented by spheres. Spongy tissue was composed of spongy cells of different sizes, with the final number of spheres depending on a predetermined air space-to-tissue volume ratio (0.45 for the geometry in Fig. 1a). Specifically, we first sequentially and randomly positioned spheres with an initial radius of 12 µm. If the pre-set ratio was not reached and there was not enough space to add more spheres with this radius, the radius was reduced by 10% and the air space was filled using smaller spheres. This process was iterated until the final, pre-set ratio was reached. Inside the epidermis, palisade and initial spongy cells, a cell wall was defined as a 1 µm-thick layer. A vacuole was represented by an ellipsoid, positioned in the center of mesophyll cells. Cytosol filled the remainder of the cell volume (Govaerts *et al.*, 1996).

In addition, we extended the work of Govaerts *et al.* (1996) by modeling chloroplasts as ellipsoids along cell walls in our geometry, instead of as a continuous layer inside a mesophyll cell next to the wall in Govaerts *et al.* (1996). Ellipsoid chloroplasts (number and dimensions given in Table 1) were distributed along the cell walls of the palisade and spongy cells. The centers of those ellipsoids were selected from a set of points that were pre-generated and uniformly distributed along the wall with a pre-set minimum distance between one another. This distance was set to ensure that the chloroplasts placed inside cells would not intersect. For the default model, the chloroplasts position was selected randomly from this point set (Fig. 1a). When chloroplast face and profile positions were modeled, only points around the cell top/bottom and around the middle region of the cells, respectively, were selected (Fig. 1b, c).

**Table 1.** *Structural parameters used to reconstruct a generic dicot leaf* For the chloroplast size in palisade and spongy cells, a, b and c represent the length of three semi-principle axes. For the spongy cells, since different sizes of cells are utilized to reconstruct the spongy tissue with a defined fraction of airspace, only the maximum cell radius and cell wall thickness are listed here.

**Tissue**	**Characteristic**	**Value**
Epidermis	Cell radius	12.5 μm
	Cell oblateness	0.3
	Cell wall thickness	1 μm
Palisade	Cell radius	7.5 μm
	Cell height	70 μm
	Cell wall thickness	1 μm
	Chloroplast number per cell	172
	Chlorophyll concentration	0.7×10^5^ g m^−3^
	Chloroplast size (axis *a*×*b*×*c*)	2 μm×1.35 μm×0.4 μm
	Vacuole radius	5.4 μm
	Vacuole height	65.8 μm
Spongy	Maximal cell radius	12 μm
	Maximal cell wall thickness	1 μm
	Fraction of airspace	0.45
	Chloroplast number per cell	69
	Chlorophyll concentration	10^5^ g m^−3^
	Chloroplast size (axis *a*×*b*×*c*)	1.8 μm×1.8 μm×0.5 μm
	Vacuole radius	9.6 μm
	Tissue thickness	70 μm

Chloroplast size and number in palisade and spongy tissues (Table 1) were determined based on previous observations on chloroplast size, number and surface area coverage (Honda, 1971; Evans, 1983; Araus *et al.*, 1986; Evans & von Caemmerer, 1994; Wise and Hoober, 2007). In this study, the coverage of chloroplast surface relative to cell wall surface was calculated as the ratio between the sum of the projected area of all chloroplasts and the surface area of all mesophyll cell wall. The coverage can be controlled by changing the number of chloroplasts and the size of each chloroplast. In this study, we used a chloroplast coverage of around 45% for most analyses. We also conducted a sensitivity analysis on the influence of modifying the chloroplast coverage on the leaf internal light environment. When we simulated different chloroplast positions, the coverage of chloroplast to cell surface was controlled to be the same.

To model the light scattering property of BSEs, we first reserved a defined proportion of leaf area in both palisade and spongy tissue for BSE cells (Fig. 1d). BSE cells were represented by spheres without chloroplasts (sphere radius and wall thickness are given in Table 1). In this model, the structures and optical properties of bundle sheath and vein were simplified as BSEs. Fig. 1d illustrates the leaf structure where BSEs occupied 20% of the total leaf volume. When 40% of leaf volume was occupied by BSEs, an additional layer of BSEs was added. The method for placing BSE cells in this reserved space was the same as that for spongy cells.

To compare the optical properties between palisade tissue and spongy tissue, the palisade layer and spongy layer were separated from the whole leaf shown in Fig. 1a and the light absorption profiles of both tissues were simulated separately under either direct or diffuse incident light. Since the thickness and chlorophyll content of these two tissues were equal, the difference of the predicted light absorption profiles for these two tissues can only be due to anatomical differences. Therefore these simulated absorption profiles can be used to gain insight into the effect of anatomy on the optical properties of palisade and spongy tissues.

### The Monte Carlo ray tracing algorithm

With the leaf anatomy described above, the direct light was mimicked by collimated rays perpendicular to leaf surface, while the diffuse light was mimicked by rays with random directionality. The reflectance and transmittance on each interface of two different media, such as between intercellular air and cell walls, or between cytosol and chloroplasts, can be calculated using Fresnel equations with the refractive indexes listed in Govaerts *et al.* (1996) (Supplementary Table S1 at *JXB* online). For each component of a cell, i.e. cell wall, chloroplast, cytosol and vacuole, absorption was calculated by the Beer–Lambert law. The specific absorption coefficients for these components under different wavelengths used in this model were the same as those used by Govaerts *et al.* (1996) (Supplementary Table S1). The chlorophyll concentration of each palisade and spongy chloroplast can be set arbitrarily, and in the default model (Table 1) it was estimated based on three assumptions. Firstly, the chlorophyll concentration per unit tissue volume was assumed to be relatively uniform across leaf depth (Vogelmann & Evans, 2002). Secondly, the chlorophyll content of the modeled typical dicot leaf was taken to be 50 µg cm^−2^ (Hosgood *et al.*, 1994). Thirdly, the chlorophyll concentration in the chloroplasts of palisade or spongy cells was the same. Then together with the size and number of chloroplasts in palisade and spongy mesophyll cells, the chlorophyll concentration per chloroplast volume was calculated (Table 1).

**Fig. 2.**
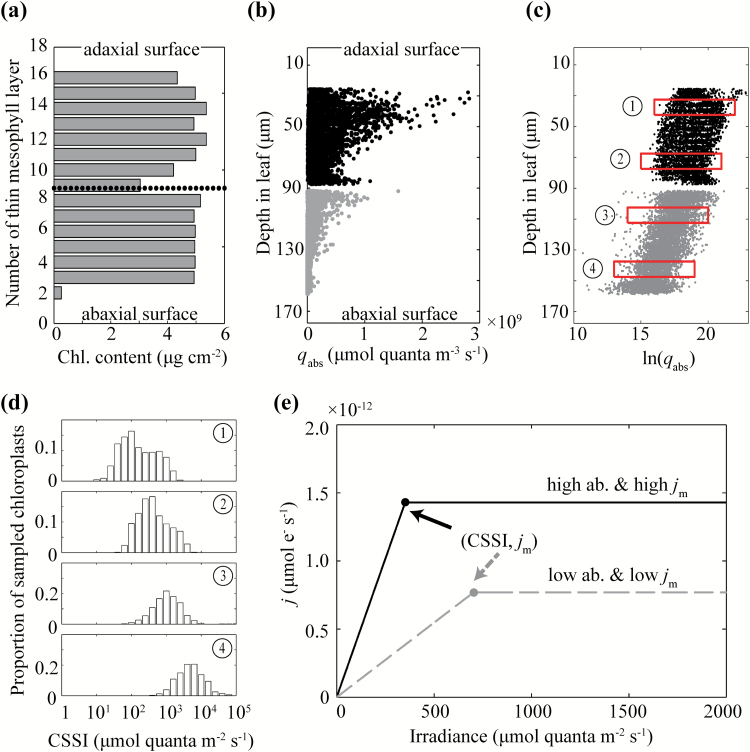
(a) The chlorophyll distribution of the modeled leaf from the adaxial surface to the abaxial surface. Each thin mesophyll layer represents 1/18th of the total leaf thickness. Each bar represents the chlorophyll content estimated in a particular thin mesophyll layer. The dotted line represents the boundary between palisade and spongy mesophyll. (b) The distribution of volumetric photon absorption (*q*_abs_) across the leaf. Each dot represents one chloroplast. The depth in the leaf is measured from the upper to the lower side of the leaf. (c) The log-transformed quantity of what was shown in (b). Red boxes indicate the four sampling regions for the four histograms shown in (d). These histograms show the distribution of the chloroplast specific saturating irradiance (CSSI) calculated using Eqn 3. CSSI is the minimum incident light intensity on the surface of the leaf needed to saturate a specific chloroplast inside the leaf. Each bar represents the proportion of chloroplasts with a certain range of CSSI in a sampling region. (e) Light curves for two different chloroplasts, one of which has high absorptance of light (ab) and high *j*_m_, the other has low absorptance and low *j*_m_.

In the default model, the chlorophyll distribution was assumed to be uniform across the leaf (Fig. 2a); however, it has been reported that the chlorophyll concentration in the palisade tissue is higher than that in the spongy tissue (Terashima and Saeki, 1983; Cui *et al.*, 1991), and that the chlorophyll content is highly heterogeneous along the leaves (Xiong *et al.*, 2015). Therefore we also examined the effect of variation in chlorophyll concentration between palisade and spongy chloroplasts on the internal light environment. The total leaf chlorophyll content was kept constant during this analysis.

Ideally, during a ray-tracing process, each ray splits into two rays after hitting an interface between two cellular components. One of these two rays represents the reflected ray and another represents the refracted ray. As a result, during the simulation, the number of light rays would increase exponentially. To limit the amount of computation power necessary, a Monte Carlo approach was applied. Specifically, only the reflected ray or the refracted ray was followed during the next step in the ray-tracing process. In either case, this ray carried the total light energy of the incident ray. Based on the Fresnel equations, we determined whether a generated ray was reflected or transmitted depending on a probability proportional to light intensity.

A box fit to the reconstructed leaf geometry shown in Fig. 1 was used as the boundary of the ray tracing algorithm, and the total energy of rays that hit the top or bottom face was recorded as the reflectance and transmittance of the leaf, respectively. If one ray hit the left or right edge of the box, then a circular boundary condition (Song *et al.*, 2013) was applied, i.e. the starting point of that ray was mirrored to the opposite face on the boundary box and its direction was kept unchanged. The source code is available from authors upon request.

### Links between the light profile and the light response curve of the electron transport rate

The photosynthetic electron transport rate is limited by the absorbed light flux density, electron transport capacity, and availability of NADP^+^ and ADP. The electron transport rate in the presence of saturating NADP^+^ and ADP is denoted as potential electron transport rate (*J*) (Buckley and Farquhar, 2004). To avoid confusion of the term ‘potential electron transport rate’ with the term ‘maximum electron transport rate’ in literature, hereafter in this paper we directly use electron transport rate to represent the electron transport rate in the presence of saturating NADP^+^ and ADP.

A hyperbolic equation for the light response curve of *J* is often used (Eqns 1 and 2 in Farquhar & Wong, 1984; von Caemmerer, 2000; Buckley and Farquhar, 2004). Here, instead of applying these equations to the whole leaf, we distributed the whole leaf electron transport capacity (*J*_m_) over all chloroplasts (*j*_m_). This *j*_m_ was further used to calculate the electron transport rate for each chloroplast (*j*) at a particular light level. The whole-leaf electron transport rate (*J*) was then calculated as the integral of the electron transport rates of all chloroplasts in a leaf. In this paper, upper case and lower case symbols are used to represent properties of leaf and chloroplast respectively. For example, *J* is used to represent the electron transport rate of the leaf, while *j* is used to represent the electron transport rate of a chloroplast; *J*_m_ is used to represent the electron transport capacity of a leaf and *j*_m_ is used to represent the electron transport capacity of a chloroplast.

In the default model, *J*_m_ was set to 120 µmol m^−2^ s^−1^, and *j*_m_ was assumed to be proportional to the chlorophyll content of each chloroplast leading to a uniform distribution. We also simulated scenarios where *j*_m_ was non-uniformly distributed since it has been reported that the profile of *j*_m_ is proportional to the internal absorption gradient (Evans and Vogelmann, 2003; Buckley and Farquhar, 2004). In this case, we assumed that *j*_m_ was linked to light absorption by a specific chloroplast, which was in turn determined by the light absorbed by a cell and the number of chloroplast in the cell. This *j*_m_ profile is hereafter termed the proportional *j*_m_ profile. This profile is essentially equivalent to the result of an optimization of electron transport capacity at the cellular level, as predicted by the model of Farquhar (1989). Considering that chloroplasts can relocate within mesophyll cells, we did not consider this optimization of chloroplast electron transport capacities within a single cell.

For each chloroplast, *j* was calculated by the following equations:

$$\theta {\left(j^{k}\right)}^{2}-\left(j_{\text{i}}^{k}+j_{\text{m}}^{k}\right)j^{k}+j_{\text{i}}^{k}j_{\text{m}}^{k}=0$$ (1)

$$j_{\text{i}}^{k}=I\times S\times \alpha^{k}\times \left(1-f\right)/2$$ (2)

where *k* is the index of a specific chloroplast, *θ* is a convexity index between 0 and 1, *j* ^k^ is the electron transport rate (μmol e^−^ s^−1^) of the *k*th chloroplast, *j*_i_^k^ is the light limited rate of PSII electron transport (μmol e^−^ s^−1^) of the *k*th chloroplast under a particular incident irradiance, *j*_m_^k^ is the electron transport capacity (μmol e^−^ s^−1^) of the *k*th chloroplast, *I* is the incident irradiance (μmol photons m^−2^ s^−1^) on a leaf, *S* is the modeled leaf area (m^2^), *α* ^k^ is the light absorptance of the *k*th chloroplast in the modeled leaf, *f* is fraction of absorbed photons that do not drive electron generation (~0.15, Evans 1987) and 2 is used as the denominator in Eqn 2 because each photosystem absorbs half of the available light.

The convexity index *θ* was assumed to be 1 considering that the measured *θ* of chloroplast suspension is very close to 1 (Terashima and Saeki, 1985; Ögren and Evans, 1993). In this case, the hyperbolic Eqn 1 degenerates to a simple form, i.e. *j* ^k^=min{*j*_i_^k^,*j*_m_^k^}, indicating that a specific chloroplast in the leaf is light saturated if and only if *j*_i_ is larger than *j*_m_. For convenience in analysing chloroplast light saturation we defined the chloroplast specific saturating irradiance (CSSI) as the minimum incident light intensity on the surface of the leaf needed to saturate a specific chloroplast inside the leaf (Fig. 2e):

$$j_{\text{m}}^{k}={\text{CSSI}}^{k}\times S\times \alpha^{k}\times \left(1-f\right)/2$$ (3)

## Results

### Model simulation

Light absorptance by each chloroplast was predicted using the ray-tracing algorithm. Since chloroplasts have different sizes in our model, chloroplast light absorptance was normalized by chloroplast volume and denoted as volumetric light absorptance *q*_abs_ (µmol quanta m^−3^ s^−1^). Fig. 2b shows *q*_abs_ at different depths inside a leaf and each dot in the figure represents one chloroplast in the palisade or spongy mesophyll cells. This simulation used direct (i.e. parallel light rays) blue light as incident light source with a PFD on the leaf surface of 1000 µmol m^−2^ s^−1^. We plotted the natural logarithm of the *q*_abs_ to facilitate comparisons with the Beer–Lambert law, which predicts an exponential decrease of energy density with depth (Fig. 2c). Although our results show that the logarithm of *q*_abs_ decreased linearly with depth, the light environment varied not only with depth but also within the same layer, especially in the spongy tissue. To describe and investigate such paradermal variations in light absorption, we sampled four thin sections of chloroplasts in the leaf (red box in Fig. 2c) and plotted the corresponding histograms of chloroplast specific saturating irradiance (CSSI), i.e. the minimal incident irradiance required to saturate a specific chloroplast (Fig. 2d).

### Leaf light reflectance, transmittance and absorptance spectrum

**Fig. 3.**
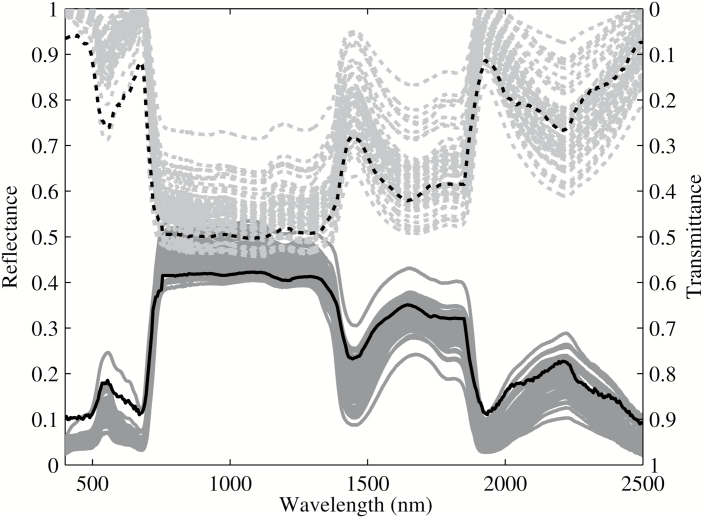
Model-predicted reflectance (black solid line) and transmittance (black dotted line) spectrum. The difference between the reflectance and the transmittance represents leaf absorptance. Experimental data (gray solid lines and gray dotted lines) were measurements on 64 dicot leaves covering 48 species and were collected from the LOPEX93 database (Hosgood *et al.*, 1994).

Here, we first show that our simulated reflectance, transmittance and absorptance spectra are representative for the experimentally measured spectra from the LOPEX dataset, which is widely used by the remote sensing community (Hosgood *et al.*, 1994). We selected all measured spectra on fresh dicot leaves (64 leaves covering 48 species) and plotted these together with our simulated spectra (Fig. 3). Results show that our model predicted a very similar shape of leaf reflectance and transmittance spectra for incident wavelengths between 400 and 2500nm. The absorptance spectrum in the visible wavelength region (400–700nm) is mainly determined by leaf pigments, whereas the absorptance in the near-infrared region (800–1300nm) is related to leaf-structure, and the absorptance in the infrared region (1400–2500nm) is dependent on water (Jacquemoud and Baret, 1990).

### Leaf internal light gradients under direct and diffuse incident light

**Fig. 4.**
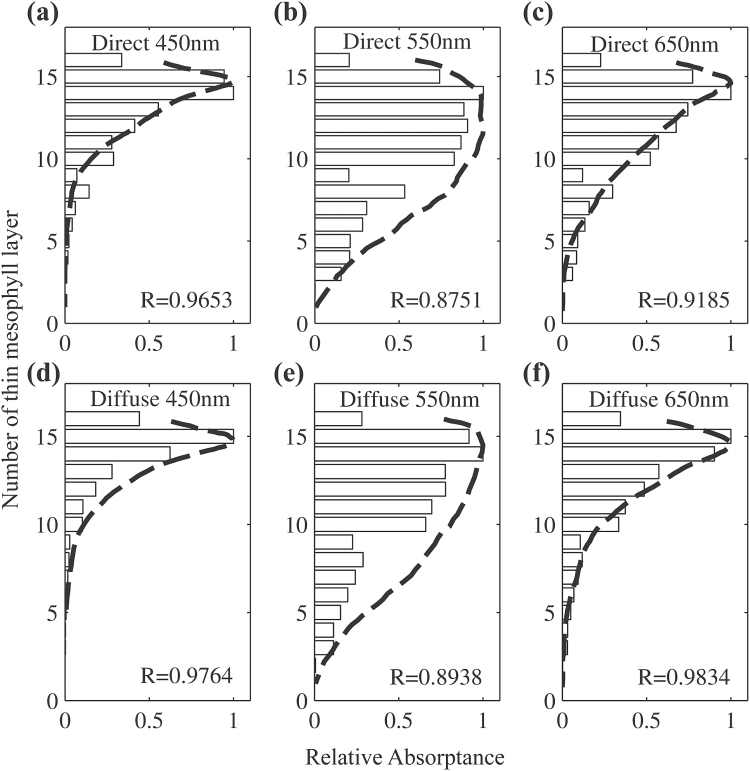
Simulated relative light absorptance is shown using bars for red, green and blue direct (a–c) or diffuse (d–f) light. A thin mesophyll layer on the *y*-axis represents 1/18th of the total thickness of the leaf as previously defined (Fig. 2a). The *x*-axis represents the relative absorptance, which is defined as the ratio of the absorptance in a thin mesophyll layer to the maximal absorptance among all thin mesophyll layers, following Brodersen and Vogelmann (2010). Experimental results of *Antirrhinum majus* from Brodersen and Vogelmann (2010) are indicated by the dashed lines.

Next we compared simulated light absorptance profiles to measured profiles from *Antirrhinum majus* (snapdragon) leaves from Brodersen and Vogelmann (2010). Both simulations and experiments were conducted for direct and diffuse light under blue, green and red wavelengths. In this analysis, the leaf was divided into 18 thin mesophyll layers with the same thickness, and light absorptance by each thin layer was determined and normalized to the maximum absorptance among all layers. We also classified the absorptance of all chloroplasts into 18 layers according to their depth in the modeled leaf and calculated the expected light absorption profiles. The normalized absorptance of each thin mesophyll layer from experiment and simulation was denoted as relative absorptance and compared in Fig. 4. Under direct or diffuse blue, green and red incident light, our simulated absorption profiles were consistent with the experimental measured profiles with correlation coefficients all higher than 0.87 (Fig. 4). Some of the predicted and measured light profiles at 650nm even showed a correlation coefficient of 0.98 (Fig. 4f). Moreover, in both simulation and experiments, the internal light gradient was steepest under blue incident light and the gradient was relatively uniform under green incident light. Simulation studies also showed a steeper gradient under diffuse incident light compared with the gradient under direct light with the same wavelength, which was also observed in experimental profiles (Fig. 4). Therefore we concluded that our model can simulate typical internal light gradients for incident light with different wavelengths and light directions.

### Optical properties for palisade and spongy mesophyll cells

**Fig. 5.**
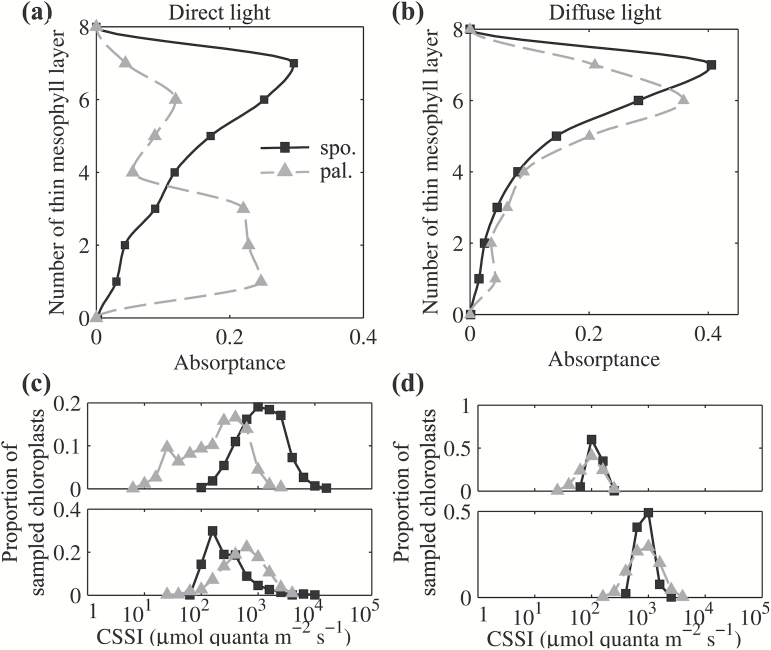
Leaf internal light profile in palisade tissue (gray dotted lines) and spongy tissue (black solid lines) of the same thickness and with equal chlorophyll content under direct blue light (a) or diffuse blue light (b). Each thin mesophyll layer represents 1/18th of the total leaf thickness. The corresponding distribution of CSSI under direct blue light (c) or diffuse blue light (d) in sampling regions is the same as for the whole leaf model in Fig. 2c. There are two sampling regions in both palisade and spongy tissue. The upper panels in (c) and (d) compare the histograms for upper sampling region in palisade and spongy tissue, and the lower panels compare the lower ones.

We further examined whether the model simulation is consistent with previous observations of optical properties of palisade and spongy tissues. To this end, we extracted two tissue structures from the leaf geometry in Fig. 1a: one composed of only the palisade tissue without epidermis and spongy cells, and the other composed of only the spongy tissue without epidermis and palisade cells. Both structures had the same thickness and chlorophyll content, and allowed therefore the comparison of the effect of anatomical structure (palisade or spongy) on leaf optical properties. The results of the ray tracing showed that, consistent with earlier reports (Vogelmann & Martin, 1993; Brodersen and Vogelmann, 2010), the columnar palisade cells minimized light scattering and enabled light to penetrate deeper into a leaf (Table 2, Fig. 5a). By contrast, the spherical spongy cells were more effective in scattering light and thus maximized light absorptance for the whole leaf (Table 2). Furthermore, our simulations show that in the isolated palisade mesophyll layers the leaf internal light gradients differed dramatically depending on whether the incident light was diffuse or direct. Such differences were, however, not observed for isolated spongy tissue (Fig. 5). These observations are consistent with experimental literature (Vogelmann and Martin, 1993), which shows that the light facilitation effect of the palisade was much stronger under direct light compared with diffuse light.

**Table 2.** Simulated reflectance, absorptance and transmittance in isolated palisade and spongy tissue under red, green, blue and direct or diffuse incident light

Parameter	Direct			Diffuse		
	450 nm	550 nm	650 nm	450 nm	550 nm	650 nm
Palisade tissue						
Reflectance	0.015	0.047	0.023	0.035	0.063	0.039
Absorptance	0.530	0.310	0.423	0.925	0.641	0.825
Transmittance	0.455	0.643	0.554	0.041	0.296	0.136
Spongy tissue						
Reflectance	0.021	0.109	0.053	0.042	0.152	0.082
Absorptance	0.940	0.553	0.768	0.938	0.619	0.811
Transmittance	0.039	0.338	0.179	0.020	0.229	0.107

### Focusing of light by lens-shaped epidermal cells

**Fig. 6.**
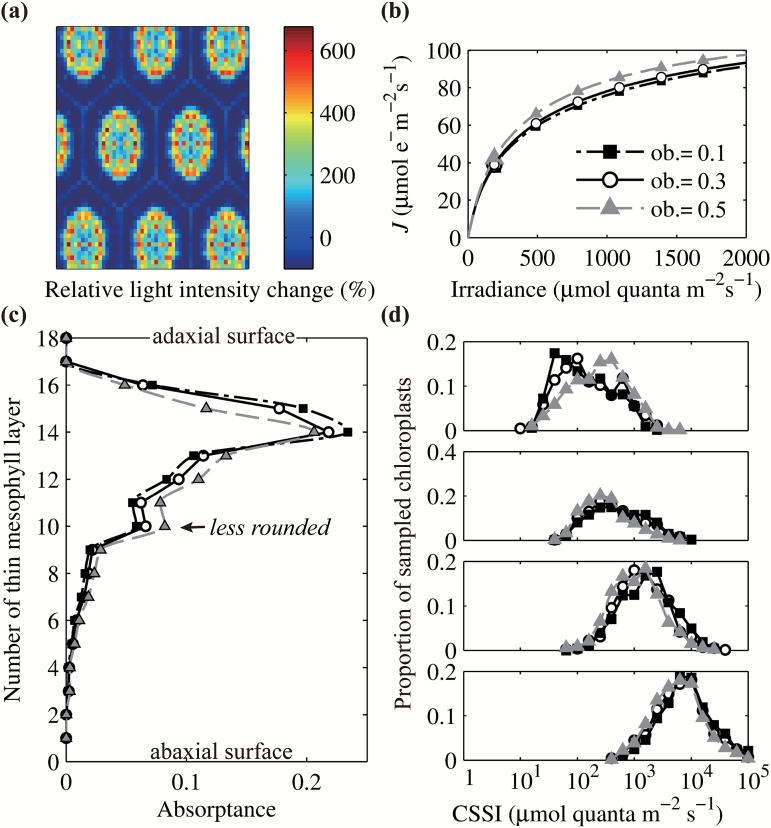
(a) Distribution of light after transmission through epidermal cells with an oblateness of 0.3 under direct incident light. Different colors represent the relative change, which is the percentage change of local incident PFD relative to the incident PFD on the leaf surface. (b) The effect of epidermal cell oblateness on the light curves. Epidermal cells with bigger oblateness means they are less rounded. (c) The effect of epidermal cell oblateness on the distribution of light absorption across leaf depth under direct blue light. Each thin mesophyll layer represents 1/18th of the total leaf thickness. (d) The effect of epidermal cell oblateness on the distribution of CSSI (defined in the legend of Fig. 2) in the four previously defined (Fig. 2c) sampling regions. Open circles represent results with an epidermis oblateness of 0.3, gray triangles represent results with an oblateness of 0.5 and black squares represent results with an oblateness of 0.1.

To quantitatively examine the focusing effect of lens-shaped epidermal cells (Haberlandt, 1914; Vogelmann *et al.*, 1996), we first simulated the distribution of light energy transmitted through the epidermis. Because the epidermis modeled here contained no pigments and the small difference in refractive indexes of cell wall and cytosol under different wavelengths was ignored, the predicted light transmittance through the epidermis did not depend on the incident light wavelength. Direct light transmitted through the epidermis was much more focused than diffuse light (Fig. 6a, Supplementary Fig. S1). At some locations, 20 µm beneath the leaf surface, direct incident light resulted in a PFD that was almost 7 times higher than the incident PFD. The focusing effect was much weaker for diffuse light, with the maximal increase in PFD being around 2.5-fold (Supplementary Fig. S1a). It is important to emphasize that total energy was being conserved, i.e. the more energy was being focused on a small region of a leaf, the larger the regions were, where the light intensity was reduced (Fig. 6a, Supplementary Fig. S1). We further compared the focusing effects under different epidermal cell oblateness, with an oblateness of 0.5 representing a more flattened cell and 0.1 representing a more round or spherical cell. Our results show that a more flattened epidermis resulted in a reduced focusing effect (Supplementary Fig. S1) and rounder epidermal cells resulted in a more heterogeneous leaf internal light environment.

We also examined the impact of varying oblateness of epidermis on the light absorption inside a leaf. Results show that for blue light, the absorptance of the first three thin mesophyll layers (each representing 1/18th of the total thickness of the leaf) was increased by 12.4%, 11.2% and 7.3%, respectively, when oblateness was decreased from 0.3 to 0.1 (i.e. when the cells became more rounded). This suggests that more rounded cells in the epidermis resulted in a more uneven absorptance by the underlying mesophyll cells (Fig. 6c). In other words, more light was focused on some chloroplasts and was being absorbed instead of penetrating deeper inside the leaf. This was also consistent with the shift in the peak of the CCSI histogram for chloroplasts in the upper layers of the leaf (Fig. 6d), which means that a larger fraction of the chloroplasts in the leaf absorbed more light when the epidermal cells were more rounded. In contrast, the absorptance of the lower layers in the leaf was increased when the epidermal cells were less rounded (Fig. 6c), in other words, the absorption profile became more uniform. For direct, green and red light the effect of the epidermal cell oblateness on the internal light environment was comparable to that for the blue light shown above (Supplementary Fig. S2). This impact on the leaf internal light environment by oblateness of the epidermal cells also influenced the estimated electron transport rate (*J*) (Fig. 6b). Under a uniform profile of *j*_m_, a more uniform absorption profile resulted in a higher light-use efficiency (Fig. 6b, c). For diffuse light, the absorptance profile of the top three layers was not significantly changed by differences in the oblateness of the epidermal cells (Supplementary Fig. S2).

### The effect of bundle sheath extensions on light-use efficiency

We constructed two different leaf structures with different proportions (*ca*. 20% and 40%) of bundle sheath extensions (BSEs) occupying part of the leaf volume. Simulations were conducted to quantitatively compare the influence of BSEs on the internal light environment and light response curves by comparing these two leaf structures with a leaf structure without BSEs (Fig. 1a). In this simulation, we kept the number of chloroplasts and the chlorophyll concentrations in each mesophyll cell the same, which inevitably led to different chlorophyll contents per leaf area, i.e. 40.75 and 31.13 µg cm^−2^ for the leaf structures with respectively 20% and 40% BSE occupation.

**Fig. 7.**
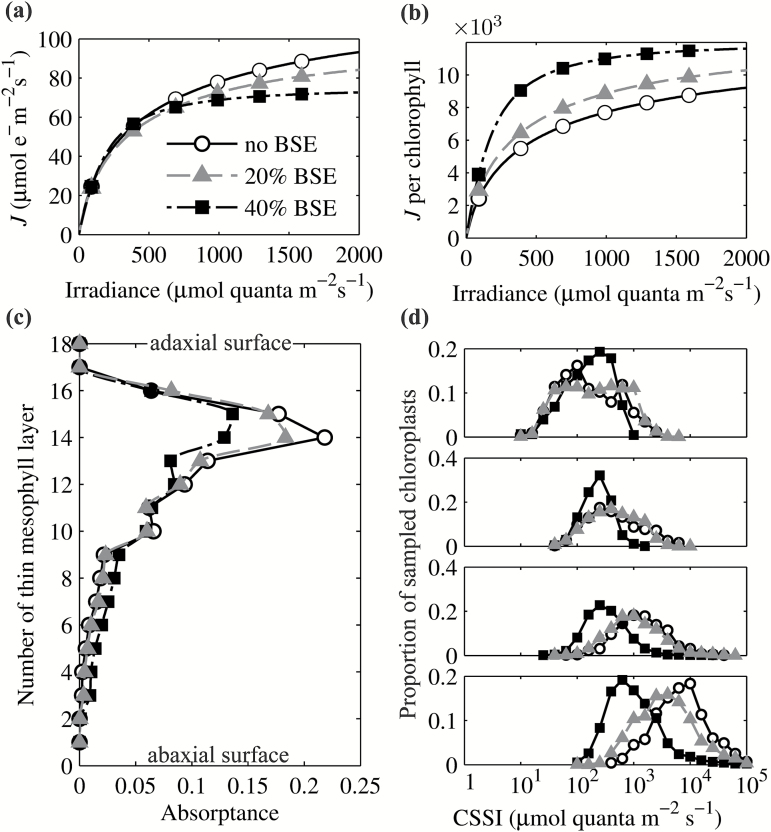
(a) The predicted leaf light curves for three modeled leaves. (b) The predicted leaf *J* per unit chlorophyll increased with an increased proportion of BSEs under all incident irradiance. (c) The effect of BSEs on the distribution of the light absorption across leaf depth under direct blue light. (d) The effect of BSEs on the distribution of CSSI (defined in the legend of Fig. 2) for the four sampling regions defined in Fig. 2c. Open circles represent results with no BSEs, gray triangles represent results with 20% BSE geometry and black squares represent results with 40% BSE geometry.

Results show that, under direct, blue light (Fig. 7c), an increase in the proportion of BSEs decreased light absorptance in the palisade layer and increased absorptance in the spongy region. Furthermore, increasing the proportion of BSEs led to a lower fraction of chloroplasts in each layer that were light saturated at higher levels of incident light intensity (Fig. 7d), which resulted in enhanced total leaf light-use efficiency, i.e. increased *J* on a leaf chlorophyll content basis (Fig. 7b), although *J* (per unit area) was decreased due to the decrease of leaf *J*_m_ (Fig. 7a). The same observation holds for green and red direct light or diffuse light (Supplementary Fig. S3).

### Chloroplast arrangement

**Fig. 8.**
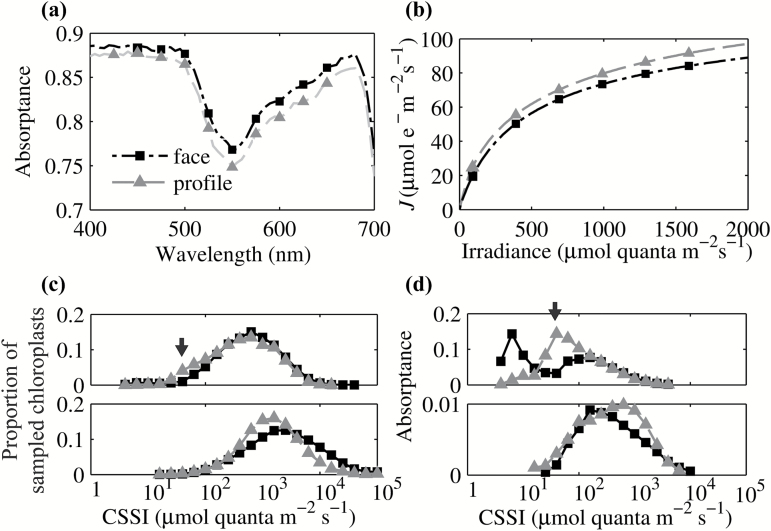
(a) The effect of chloroplasts positions on the leaf light absorptance spectrum between 400 and 700nm under direct light. (b) The predicted effect of chloroplast positions on light curves under direct blue light. (c) The effect of chloroplast arrangement on the distribution of CSSI in palisade tissue (top) and spongy tissue (bottom) under direct blue light. Black squares represent results for chloroplasts in face position and gray triangles represent results for chloroplasts in a profile position. (d) Corresponding distribution of light absorptance of (c), i.e. the sum of the absorptance of all chloroplasts with the same CSSI in (c) (the definition of CSSI is in the legend of Fig. 2). Black arrow indicates the absorptance reallocated under the two chloroplast positions.

Chloroplasts in mesophyll cells were modeled in either a face position, i.e. arranged along the upper or lower sides of the cell (Fig. 1b), or in a profile position, i.e. arranged along the side walls (Fig. 1c). When chloroplasts were arranged in the face position, our results suggest that leaf absorptance for light between 400 and 700nm was increased by only 2–3% relative to a leaf with chloroplasts in the profile position (Fig. 8a). However, this enhanced light absorptance did not necessarily lead to increased *J*. For example, using a wavelength of 450nm, we predicted that *J* of a leaf with chloroplasts in the face position was around 5 μmol m^−2^ s^−1^ less compared with the *J* of a leaf with chloroplasts in the profile position under a large range of light irradiances (Fig. 8b).

Since the chloroplast distribution within the leaf differs significantly between face and profile positions, i.e. in each paradermal section, the number of chloroplasts differs, we cannot compare the CSSI at the level of individual paradermal layers as was done in Fig. 2d. Instead we compared the CSSI between face and profile positions in palisade and spongy tissues as a whole (Fig. 8c). Results show that when chloroplasts are arranged in a profile position, the number of chloroplasts that were light saturated at an incident PFD around 10 µmol m^−2^ s^−1^ was slightly smaller than when chloroplasts were arranged in a face position (Fig. 8c). A corresponding distribution of light absorption (Fig. 8d), i.e. the sum of the absorptance of all chloroplasts with the same CSSI, showed more clearly that a large proportion of energy was reallocated from chloroplasts with low CSSI in the face position to chloroplasts with intermediate CSSI in the profile position under direct blue light (Fig. 8d).

### The effect of chlorophyll distribution on the light absorption profile

Besides the influence of incident light properties and anatomical features described above, the simulated light absorption profile was also influenced by the vertical distribution of chlorophyll (Supplementary Fig. S4). With 20% of the leaf chlorophyll reallocated between palisade and spongy tissues, the absorption gradient under direct blue light only changed slightly (Supplementary Fig. S4c), while there were greater changes in the gradient under direct green or red light (Supplementary Fig. S4d, e). However the paradermal variation of absorption was still preserved under these two different chlorophyll distributions (Supplementary Fig. S4f–h).

### The effect of the distribution of electron transport capacity across the leaf on the whole-leaf electron transport rate

**Fig. 9.**
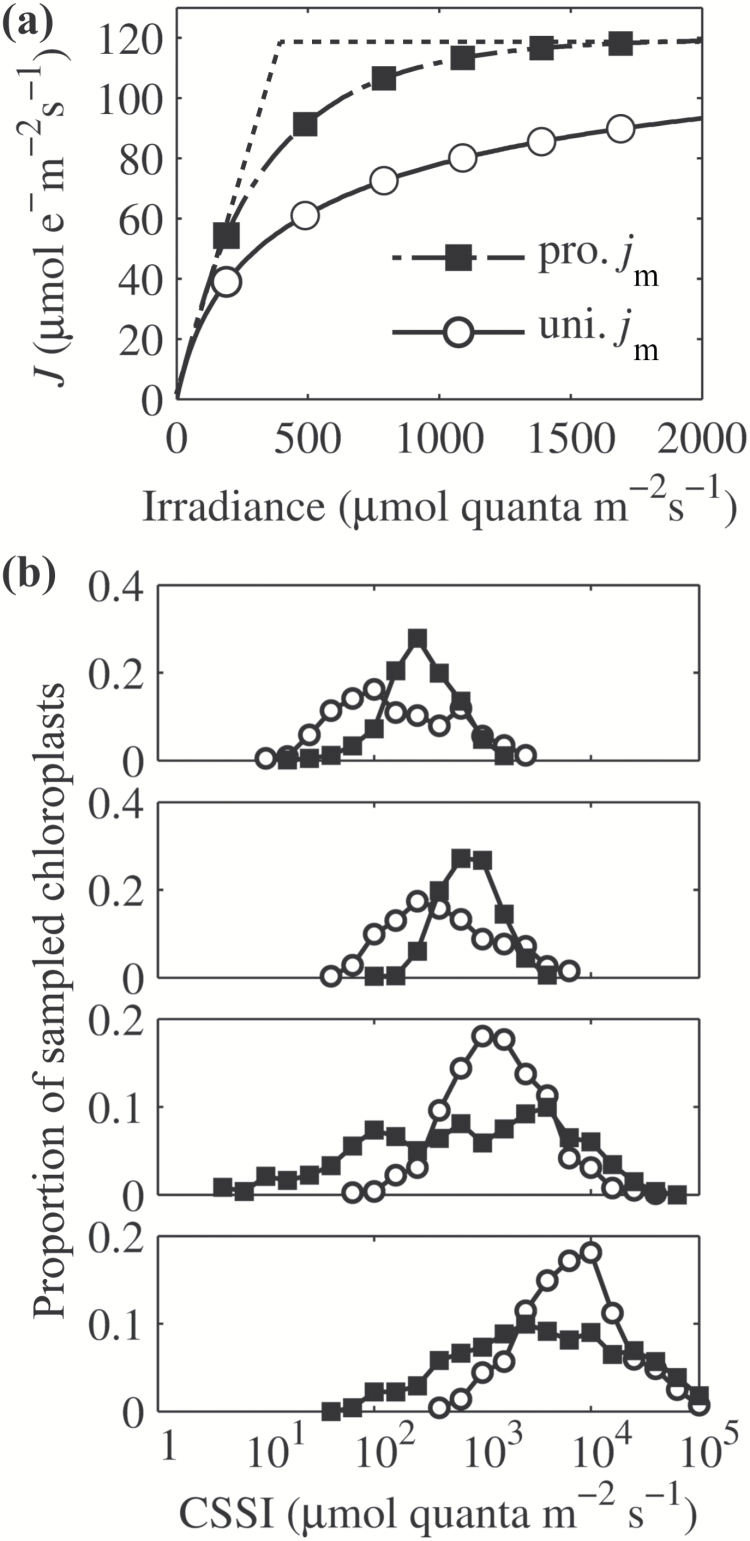
(a) The predicted light response curves of leaf electron transport rate under different *j*_m_ profiles. The dashed line represents the curve when the convexity index (*θ*) equals 1. (b) The effect of the *j*_m_ profile on the distribution of CSSI for the four sampling regions defined in Fig. 2c. The definition of CSSI is in the legend of Fig. 2. Black squares represent results under a proportional *j*_m_ profile and open circles represent the results under a uniform *j*_m_ profile.

The electron transport rate (*j*) of a chloroplast depends on both the light absorption and the electron transport capacity (*j*_m_) of that chloroplast. These two factors together affect the calculated CSSI (Eqn 3). In the default model, the profile of *j*_m_ was distributed uniformly among all chloroplasts in the leaf. As a result, the *j* of chloroplasts in the upper layers of a leaf was mostly limited by *j*_m_, while the *j* of chloroplasts at the lower layers was mostly limited by light absorption (Fig. 2d). We further simulated *J* under the assumption that *j*_m_ was non-uniform and proportional to the absorptance profile. Under this proportional *j*_m_ profile, the calculated leaf *J* was enhanced dramatically at different light intensities even though the absorption profile was the same (Fig. 9a). Moreover, the histogram of CSSI indicates that in this case, chloroplasts in the palisade cells tended to have a similar saturating irradiance, while spongy cells tended to have more chloroplasts with a relatively low CSSI (Fig. 9b).

## Discussion

### A novel three-dimensional model of leaf anatomy and internal light environment

The model presented in this study combines a ray-tracing algorithm with a programmable representation of the three-dimensional leaf anatomy. It enables researchers to not only evaluate the impact of different anatomical features in leaves, but also predict the leaf internal light environment of ‘hypothetical’ leaves with custom-designed structural and anatomical features. Govaerts *et al.* (1996) assumed that the chloroplasts form a continuous layer appressed to cell walls. As a result of this assumption the ratio between the chloroplast surface and mesophyll surface area was fixed to 1. Such an assumption may be reasonable for mesophyll cells in, for example, rice leaves, where chloroplast coverage of the cell wall is above 90% (Sage and Sage, 2009), but reported values for many other species such as spinach or wheat are around 60% (Honda *et al.*, 1971; Evans, 1983; Evans & von Caemmerer, 1994) and could be even as low as 30% for some shade-grown leaves (Araus *et al.*, 1986). Thus, the model described by Govaerts *et al.* (1996) cannot be used to represent the anatomy of many leaves and can underestimate the sieve effect, i.e. the fact that light can propagate more easily through a mesophyll cell with fewer chloroplasts (Terashima *et al.*, 2009). With this new model, chloroplasts are represented individually and separated by stretches of cytosol, which enables exploration of the effects of chloroplast properties, such as size, number, coverage, and arrangement, on the internal light gradient and *J*.

The model developed in this study was based on parameters for a generic dicot leaf and a number of comparisons between the simulation and experiment were made to evaluate our model’s performance. First, we showed that the predicted reflectance, transmittance and absorptance spectra were well within the range typically observed for many dicotyledonous species (Fig. 3). Considering that this spectrum included the photosynthetically active visible region (400–700nm), the structure-related near-infrared region (800–1300nm) and the water-related infrared region (1400–2500nm), this model can be used to predict the leaf optical properties for a broad range of wavelengths. Secondly, we found strong correlations between our model’s predictions with measured light gradients inside a leaf using direct and diffuse incident light of different wavelengths (Fig. 4). Thirdly, simulations from this model show that palisade mesophyll cells facilitate penetration of direct, but not diffuse, light; in addition, spongy mesophyll cells strongly scatter light and can increase whole-leaf light absorptance (Fig. 5, Table 2). All these are consistent with earlier observations (Brodersen and Vogelmann, 2010).

Though the model presented in this paper aimed to give a realistic representation of leaf anatomical features, a number of simplifications were still made. First, this model assumed that chlorophyll *a* and chlorophyll *b* were the only light absorbing pigments; other pigments, such as zeaxanthin and carotene, were not included. Secondly, the scattering of light by organelles such as mitochondria, or biominerals (Gal *et al.*, 2012), was not included in the present model. Thirdly, considering that the wavelength of light and the size of some cellular components in plant cells are of the same magnitude, the Fresnel equations may not perfectly describe the light propagation and the effect of scattering could be greater than predicted (Ho *et al.*, 2016). Fourthly, in this model, we assumed that chlorophyll was distributed uniformly in each chloroplast, which therefore cannot be used to study the sieve effect caused by the ultrastructure of chloroplasts. Finally, the assumed ellipsoid shape of the epidermis, the columnar shape of palisade cells and the spherical shape of spongy-tissue cells are simplifications of the real leaf structure. These areas can be improved with the modeling framework presented in this study once more accurate 3D reconstruction of leaf anatomy, more accurate quantification of biochemical variation across the leaf, and improved methods for measuring the light environment inside the leaf are available. These simplifications would potentially lead to an underestimation of the leaf absorptance and overestimation of the reflectance and transmittance slightly in the visible region (Fig. 3). This can be improved by the adoption of a more realistic tissue structure and by taking into account additional scattering properties in the ray tracing process (Watté *et al.*, 2015). Nevertheless, the ability of the model to predict the currently available data on the leaf internal light gradient, optical properties and observed optical phenomena suggests that it can already be used as an exploratory tool to study the impact of many anatomical features on the internal light environment and resulting electron transport rates.

### Physical mechanisms underlying the heterogeneity of the leaf internal light environment

The light levels inside the leaf are highly heterogeneous both in the transverse and in the paradermal direction (Fig. 2). This heterogeneity exists in simulations with different anatomical features, such as changes in the distribution of chlorophyll (Supplementary Fig. S4) and chloroplast number (Supplementary Fig. S5), and under different incident light properties, such as different wavelengths and incident light directions (Supplementary Figs S2–S4 and S6). Among all these factors influencing the internal light environment, the properties of the incident light have a greater impact on both the vertical light gradient and the paradermal variation than the anatomical parameters that we examined (Fig. 4 and Supplementary Fig. S1 *versus* Figs 5–8). These light properties also greatly influence the focusing effect of epidermis (Fig. 6a, Supplementary Fig. S2). Furthermore, among the anatomical parameters, the proportion of palisade, spongy mesophyll and BSEs showed greater influence on the internal light environment, compared with variation in subcellular structure, such as the number and location of chloroplasts (Figs 5, 7 and Supplementary Fig. S4 *versus* Fig. 8 and Supplementary Fig. S5). An increased proportion of BSEs decreased the proportion of chloroplasts that were light saturated under high incident light levels (Fig. 7d), which correspondingly increased *J* per unit chlorophyll (Fig. 7b).

Most of the predicted variation in the internal light environment can be explained by three mechanisms, i.e. the sieve effect, the detour effect and the focusing effect. The sieve effect results in a decreased absorptance because of the non-uniform distribution of pigments; the detour effect results in an increased absorption due to lengthening of the light path (Osborne & Raven, 1986; Terashima *et al.*, 2009); the focusing effect results in less uniform paradermal distribution of light (Fig. 6a, Supplementary Fig. S2). The focusing effect was related to the flattening (oblateness) of the cells in the upper epidermis (Fig. 6). The absorptance of the upper 17% of the thickness of the leaf increased when epidermal cells were more rounded because a greater proportion of energy was focused on regions with chloroplasts than on regions without chloroplasts (Fig. 6). The sieve effect was strongly related to the number of chloroplasts in a leaf (Supplementary Fig. S5). Specifically, fewer chloroplasts resulted in less coverage of the cell surface with chloroplasts, and this would allow light to penetrate deeper in the leaf (Supplementary Fig. S5). The impact of the detour effect is reflected in the distribution of CSSI under incident light of different wavelengths. The distribution was more heterogeneous under blue light (Fig. 2d) compared with that under green (Supplementary Fig. S6a) or red light (Supplementary Fig. S6b). This can be explained by the fact that green and red light are less effectively absorbed by chlorophyll, so compared with blue light, scattering of these wavelengths increases and the path length of light propagation through the leaf is longer, i.e. the detour effect increases (Terashima *et al.*, 2009).

### The effects of chloroplast arrangement on the leaf light-use efficiency

The effect of light intensity on chloroplast arrangement has been known for over a century (Senn, 1908). This has been regarded as a response of plants to avoid the potentially negative impact of photoinhibition (Kasahara *et al.*, 2002; Wada, 2013). Here we only modeled two arrangements (face position and profile position), although chloroplasts can take different positions in different cell layers. For example, Terashima and Hikosaka (1995) showed that chloroplasts in the upper layers of the leaf could be in the profile position while chloroplasts in the lower layers could arrange in a face position. Nevertheless we show that these two arrangements already influence the simulated electron transport rate even under non-photoinhibitory conditions (Fig. 8b). It is worth noting here that the chloroplast profile position can also lower the coverage of chloroplast surface to cell surface in Arabidopsis (Tholen *et al.*, 2008) and hence affects the mesophyll conductance of CO_2_ diffusion and leaf photosynthesis.

The calculated *J* based on the predicted internal light environment was higher when chloroplasts were arranged in a profile position (Fig. 8d), even though the total light absorptance was lower (Fig. 8c). This is counterintuitive since higher absorptance is expected to lead to higher photosynthesis. Careful examination of the light response curves suggests a slight enhancement of *J* for leaves with chloroplasts arranged in a face position, but only at low incident irradiance. However, when the incident light levels were higher, many chloroplasts in the face position became light-saturated (Fig. 8). So although leaf absorptance was increased by leaves with chloroplasts in the face position, more chloroplasts were experiencing light intensities above saturating levels, which cannot further increase *J*. Chloroplasts shading each other inside the mesophyll resemble the situation of leaves shading each other inside a canopy. In a canopy, a more vertical leaf position results in a higher canopy light-use efficiency (Long *et al.*, 2006), and this is analogous to the situation in the mesophyll described here, where chloroplasts in a profile position improve whole-leaf electron transport rates (Fig. 8d).

### Implications of the high level of heterogeneity of leaf internal light environment for leaf physiology

The predicted high level of light heterogeneity inside a leaf may have implications for two important leaf physiological phenomena. In this study, we show that some chloroplasts in the upper layers of a leaf already become light-saturated under incident direct blue light with an intensity of only 50 µmol m^−2^ s^−1^ (Fig. 2d). Therefore, although the majority of chloroplasts at a moderate incident light intensity, e.g. at 100 μmol m^−2^ s^−1^, are far from light-saturated, some chloroplasts can experience a high PFD due to the focusing effect (Fig. 2d). Such high PFD exposure may potentially induce photoinhibition in these chloroplasts (Long *et al.*, 1994; Ort, 2001). Furthermore, due to changes in the solar angle, movement of leaves caused by wind or other perturbations, the direction of incident light relative to the leaf surface changes frequently; consequently, chloroplasts inside a leaf may routinely experience rapidly fluctuating light levels, similar to what understory leaves experience during a day (Pearcy, 1990). The high light levels caused by the focusing effect of the epidermis and the fluctuating light conditions together can pose a significant stress for photosystems in different chloroplasts inside a leaf. In line with this, fluctuating light causes photoinhibition in both rice and Arabidopsis (Kono *et al.*, 2014; Yamori *et al.*, 2016).

Another implication of this high level of light heterogeneity, combined with differences in biochemical limitations experienced by different chloroplasts, is that it may contribute to the observed low convexity factor (*θ*) of whole-leaf light curves, the origin of which has been considered elusive (Leverenz, 1994). Considering that the measured *θ* of a chloroplast suspension is close to 1, *θ* has already been proposed to be associated with the heterogeneity of light inside the leaf (Terashima and Saeki, 1985). Our simulations show that a smaller *θ* may not only be a consequence of more heterogeneous light levels (Figs 6b, 7a and 8b), but also be related to more heterogeneous biochemical limitations between chloroplasts (Fig. 9a).

## Conclusion

The leaf ray tracing model presented here enables evaluation of the contribution of different structural and anatomical features on the internal light environment of a leaf. Furthermore, it enables the design of a particular leaf structure with a desired leaf internal light environment, and hence facilitates designing leaf anatomy for enhanced photosynthetic efficiency. As an application of this model, we used it to explore the impact of different anatomical features and properties of incident light on the internal light environment of a dicot leaf. This study demonstrates that the heterogeneous light environment inside the leaf depends on the sieve effect, the detour effect and the focusing effect. Furthermore, we showed that the chloroplast position can optimize light-use efficiency in addition to playing its well-studied role in avoiding photo-damage. Bundle sheath extensions can also influence the leaf light-use efficiency expressed on a chlorophyll basis. Simulation results further showed that even under moderate light levels incident on a leaf surface, some chloroplasts inside the leaf may experience light levels that can potentially induce photoinhibition. The high heterogeneity of the light inside a leaf and the differences in biochemical limitations between chloroplasts provides a potential mechanism for the low convexity of the light response curve.

## Supplementary data

Supplementary data are available at *JXB* online.

Figure S1. Distribution of light after transmission through epidermal cells with an oblateness of 0.3, 0.5 and 0.1 under direct and diffuse incident light.

Figure S2. The effect of epidermal oblateness on the distribution of light absorptance across leaf depth under direct green and red light and diffuse green and red light.

Figure S3. The effect of BSEs on the distribution of light absorptance across leaf depth under direct green and red light and diffuse blue, green, and red light.

Figure S4. Simulated profile of light absorption under different chlorophyll profiles.

Figure S5. Simulated profile of light absorption under different numbers of chloroplasts.

Figure S6. The distribution of CSSI in the four previously defined (Fig. 2c) sampling regions under direct green and red light and diffuse blue, green, and red light.

Table S1. Absorption coefficients and refractive indexes used in the model.

Supplementary Data

## References

[CIT0001] Araus JL Alegre L Tapia L Calafell R Serret MD 1986 Relationships between photosynthetic capacity and leaf structure in several shade plants. American Journal of Botany 73, 1760–1770.

[CIT0002] Baranoski GV Rokne J 2004 Light interaction with plants: a computer graphics perspective. Chichester, UK: Horwood Publishing.

[CIT0003] Boardman N 1977 Comparative photosynthesis of sun and shade plants. Annual Review of Plant Physiology 28, 355–377.

[CIT0004] Bone RA Lee DW Norman JM 1985 Epidermal cells functioning as lenses in leaves of tropical rain-forest shade plants. Applied Optics 24, 1408–1412. 18223728 10.1364/ao.24.001408

[CIT0005] Bornman JF Vogelmann TC Martin G 1991 Measurement of chlorophyll fluorescence within leaves using a fibreoptic microprobe. Plant, Cell and Environment 14, 719–725.

[CIT0006] Brodersen CR Vogelmann TC 2007 Do epidermal lens cells facilitate the absorptance of diffuse light? American Journal of Botany 94, 1061–1066. 21636475 10.3732/ajb.94.7.1061

[CIT0007] Brodersen CR Vogelmann TC 2010 Do changes in light direction affect absorption profiles in leaves ? Functional Plant Biology 37, 403–412.

[CIT0008] Buckley T Farquhar G 2004 A new analytical model for whole-leaf potential electron transport rate. Plant, Cell and Environment 27, 1487–1502.

[CIT0009] Cui M Vogelmann TC Smith WK 1991 Chlorophyll and light gradients in sun and shade leaves of *Spinacia oleracea* . Plant, Cell and Environment 14, 493–500.

[CIT0010] Davis PA Caylor S Whippo CW Hangarter RP 2011 Changes in leaf optical properties associated with light-dependent chloroplast movements. Plant, Cell and Environment 34, 2047–59. 10.1111/j.1365-3040.2011.02402.x 21819411

[CIT0011] Davis PA Hangarter RP 2012 Chloroplast movement provides photoprotection to plants by redistributing PSII damage within leaves. Photosynthesis Research 112, 153–61. 22695784 10.1007/s11120-012-9755-4

[CIT0012] Evans JR 1983 Photosynthesis and nitrogen partitioning in leaves of *Triticum aestivum* and related species. Ph.D. Thesis, Australian National University.

[CIT0013] Evans JR 1987 The dependence of quantum yield on wavelength and growth irradiance. Functional Plant Biology 14, 69–79.

[CIT0014] Evans JR Vogelmann TC 2003 Profiles of 14C fixation through spinach leaves in relation to light absorption and photosynthetic capacity. Plant, Cell and Environment 26, 547–560.

[CIT0015] Evans JR von Caemmerer S 1994 The relationship between CO_2_ transfer conductance and leaf anatomy in transgenic tobacco with a reduced content of Rubisco. Functional Plant Biology 21, 475–495.

[CIT0016] Farquhar GD 1989 Models of integrated photosynthesis of cells and leaves. Philosophical Transactions of the Royal Society of London. Series B, Biological Sciences 323, 357–367.

[CIT0017] Farquhar G von Caemmerer S Berry J 1980 A biochemical model of photosynthetic CO_2_ assimilation in leaves of C_3_ species. Planta 149, 78–90. 24306196 10.1007/BF00386231

[CIT0018] Farquhar GD Wong SC 1984 An empirical model of stomatal conductance. Functional Plant Biology 11, 191–210.

[CIT0019] Féret J-B François C Asner GP Gitelson AA Martin RE Bidel LPR Ustin SL le Maire G Jacquemoud S 2008 PROSPECT-4 and 5: Advances in the leaf optical properties model separating photosynthetic pigments. Remote Sensing of Environment 112, 3030–3043.

[CIT0020] Gal A Brumfeld V Weiner S Addadi L Oron D 2012 Certain biominerals in leaves function as light scatterers. Advanced Materials 24, OP77–OP83. 22290773 10.1002/adma.201104548

[CIT0021] Govaerts YM Jacquemoud S Verstraete MM Ustin SL 1996 Three-dimensional radiation transfer modeling in a dicotyledon leaf. Applied Optics 35, 6585–6598. 21127682 10.1364/AO.35.006585

[CIT0022] Haberlandt G 1914 Physiological plant anatomy. London: Macmillan.

[CIT0023] Ho QT Berghuijs HNC Watté R 2016 Three-dimensional microscale modelling of CO_2_ transport and light propagation in tomato leaves enlightens photosynthesis. Plant, Cell and Environment 39, 50–61. 10.1111/pce.12590 26082079

[CIT0024] Honda SI Hongladarom-Honda T Kwanyuen P Wildman SG 1971 Interpretations on chloroplast reproduction derived from correlations between cells and chloroplasts. Planta 97, 1–15. 24493165 10.1007/BF00388401

[CIT0025] Hosgood B Jacquemoud S Andreoli G Verdebout J Pedrini G Schmuck G 1994 Leaf Optical Properties Experiment 93 (LOPEX93). Report EUR 16095 EN. Luxembourg: European Commission.

[CIT0026] Jacquemoud S Baret F 1990 PROSPECT: A model of leaf optical properties spectra. Remote Sensing of Environment 34, 75–91.

[CIT0027] Karabourniotis G Bornman JF Nikolopoulos D 2000 A possible optical role of the bundle sheath extensions of the heterobaric leaves of *Vitis vinifera* and *Quercus coccifera* . Plant, Cell and Environment 23, 423–430.

[CIT0028] Kasahara M Kagawa T Oikawa K 2002 Chloroplast avoidance movement reduces photodamage in plants. Nature 420, 829–832. 12490952 10.1038/nature01213

[CIT0029] Knapp AK Vogelmann TC McClean TM Smith WK 1988 Light and chlorophyll gradients within *Cucurbita* cotyledons. Plant, Cell and Environment 11, 257–263.

[CIT0030] Kono M Noguchi K Terashima I 2014 Roles of the cyclic electron flow around PSI (CEF-PSI) and O_2_ dependent alternative pathways in regulation of the photosynthetic electron flow in short-term fluctuating light in *Arabidopsis thaliana* . Plant and Cell Physiology 55, 990–1004. 24553846 10.1093/pcp/pcu033

[CIT0031] Laisk A Eichelmann H Oja V 2006 C3 photosynthesis in silico. Photosynthesis Research 90, 45–66. 17131095 10.1007/s11120-006-9109-1

[CIT0032] Lee DW 1986 Unusual strategies of light absorption in rain-forest herbs. In: Givnish TJ, ed. On the economy of plant form and function. Cambridge, UK: Cambridge University Press, 105–131.

[CIT0033] Leverenz JW 1994 Factors determining the nature of light dosage response curve of leaves. In: Baker NR Boyer JR, eds. Photoinhibition of photosynthesis – from molecular mechanisms to the field. Oxford, UK: Bios Scientific Publications, 239–254.

[CIT0034] Long SP Humphries S Falkowski PG 1994 Photoinhibition of photosynthesis in nature. Annual Review of Plant Physiology and Plant Molecular Biology 45, 633–662.

[CIT0035] Long SP Zhu XG Naidu SL Ort DR 2006 Can improvement in photosynthesis increase crop yields? Plant, Cell and Environment 29, 315–330. 10.1111/j.1365-3040.2005.01493.x 17080588

[CIT0036] Nikolopoulos D Liakopoulos G Drossopoulos I Karabourniotis G 2002 The relationship between anatomy and photosynthetic performance of heterobaric leaves. Plant Physiology 129, 235–243. 12011354 10.1104/pp.010943 PMC155887

[CIT0037] Nishio J Sun J Vogelmann T 1993 Carbon fixation gradients across spinach leaves do not follow internal light gradients. The Plant Cell 5, 953–961. 12271092 10.1105/tpc.5.8.953 PMC160330

[CIT0038] Ögren E Evans JR 1993 Photosynthetic light-response curves. Planta 189, 182–190.

[CIT0039] Oguchi R Hikosaka K Hirose T 2003 Does the photosynthetic light-acclimation need change in leaf anatomy? Plant, Cell and Environment 26, 505–512.

[CIT0040] Oguchi R Douwstra P Fujita T Chow WS Terashima I 2011 Intra-leaf gradients of photoinhibition induced by different color lights: implications for the dual mechanisms of photoinhibition and for the application of conventional chlorophyll fluorometers. New Phytologist 191, 146–159. 21418065 10.1111/j.1469-8137.2011.03669.x

[CIT0041] Ort DR 2001 When there is too much light. Plant Physiology 125, 29–32. 11154289 10.1104/pp.125.1.29 PMC1539318

[CIT0042] Osborne BA Raven JA 1986 Light absorption by plants and its implications for photosynthesis. Biological Reviews 61, 1–60.

[CIT0043] Pearcy RW 1990 Sunflecks and photosynthesis in plant canopies. Annual Review of Plant Physiology and Plant Molecular Biology 41, 421–53.

[CIT0044] Pearcy RW Gross LJ He D 1997 An improved dynamic model of photosynthesis for estimation of carbon gain in sunfleck light regimes, Plant, Cell and Environment 20, 411–424.

[CIT0045] Poolman MG Fell DA Thomas S 2000 Modelling photosynthesis and its control. Journal of Experimental Botany 51, 319–328. 10.1093/jexbot/51.suppl_1.319 10938839

[CIT0046] Poulson ME Vogelmann TC 1990 Epidermal focussing and effects upon photosynthetic light-harvesting in leaves of *Oxalis* . Plant, Cell and Environment 13, 803–811.

[CIT0047] Sage TL Sage RF 2009 The functional anatomy of rice leaves: implications for refixation of photorespiratory CO_2_ and efforts to engineer C4 photosynthesis into rice. Plant and Cell Physiology 50, 756–772. 19246459 10.1093/pcp/pcp033

[CIT0048] Senn G 1908 Die Gestalts-und Lageveränderung der Pflanzen-Chromatophoren. Leipzig, Germany: Wilhelm Engelmann Verlag.

[CIT0049] Song Q Zhang G Zhu XG 2013 Optimal crop canopy architecture to maximise canopy photosynthetic CO_2_ uptake under elevated CO_2_-A theoretical study using a mechanistic model of canopy photosynthesis. Functional Plant Biology 40, 109–124. 10.1071/FP12056 32481092

[CIT0050] Takahashi K Mineuchi K Nakamura T Koizumi M Kano H 1994 A system for imaging transverse distribution of scattered light and chlorophyll fluorescence in intact rice leaves. Plant, Cell and Environment 17, 105–110.

[CIT0051] Terashima I Fujita T Inoue T Chow WS Oguchi R 2009 Green light drives leaf photosynthesis more efficiently than red light in strong white light: revisiting the enigmatic question of why leaves are green. Plant and Cell Physiology 50, 684–697. 19246458 10.1093/pcp/pcp034

[CIT0052] Terashima I Hanba YT Tazoe Y Vyas P Yano S 2006 Irradiance and phenotype: comparative eco-development of sun and shade leaves in relation to photosynthetic CO_2_ diffusion. Journal of Experimental Botany 57, 343–354. 16356943 10.1093/jxb/erj014

[CIT0053] Terashima I Hikosaka K 1995 Comparative ecophysiology of leaf and canopy photosynthesis. Plant, Cell and Environment 18, 1111–1128.

[CIT0054] Terashima I Miyazawa S Hanba Y 2001 Why are sun leaves thicker than shade leaves?—Consideration based on analyses of CO_2_ diffusion in the leaf. Journal of Plant Research 114, 93–105.

[CIT0055] Terashima I Saeki T 1983 Light environment within a leaf. I. Optical properties of paradermal sections of Camellia leaves with special reference to differences in the optical properties of palisade and spongy tissues. Plant and Cell Physiology 24, 1493–1501.

[CIT0056] Terashima I Saeki T 1985 A new model for leaf photosynthesis incorporating the gradients of light environment and of photosynthetic properties of chloroplasts within a leaf. Annals of Botany 56, 489–499.

[CIT0057] Tholen D Boom C Noguchi K Ueda S Katase T Terashima I 2008 The chloroplast avoidance response decreases internal conductance to CO_2_ diffusion in *Arabidopsis thaliana* leaves. Plant, Cell and Environment 31, 1688–1700. 10.1111/j.1365-3040.2008.01875.x 18721264

[CIT0058] Tholen D Boom C Zhu XG 2012 Opinion: prospects for improving photosynthesis by altering leaf anatomy. Plant Science 197, 92–101. 23116676 10.1016/j.plantsci.2012.09.005

[CIT0059] Ustin SL 2004 Remote sensing of environment: State of the science and new directions. In: Ustin SL, ed. Manual of Remote Sensing, Vol 4, Remote sensing of natural resources management and environmental monitoring. Hoboken, NJ, USA: John Wiley & Sons, 679–729.

[CIT0060] Ustin SL Jacquemoud S Govaerts Y 2001 Simulation of photon transport in a three-dimensional leaf: implications for photosynthesis. Plant, Cell and Environment 24, 1095–1103.

[CIT0061] Vogelmann TC Bornman JF Josserand S 1989 Photosynthetic light gradients and spectral regime within leaves of *Medicago sativa* . Philosophical Transactions of the Royal Society of London. Series B, Biological Sciences 323, 411–421.

[CIT0062] Vogelmann TC Bornman JF Yates DJ 1996 Focusing of light by leaf epidermal cells. Physiologia Plantarum 98, 43–56.

[CIT0063] Vogelmann TC Evans J 2002 Profiles of light absorption and chlorophyll within spinach leaves from chlorophyll fluorescence. Plant, Cell and Environment 25, 1313–1323.

[CIT0064] Vogelmann TC Han T 2000 Measurement of gradients of absorbed light in spinach leaves from chlorophyll fluorescence profiles. Plant, Cell and Environment 23, 1303–1311.

[CIT0065] Vogelmann TC Knapp AK McClean TM Smith WK 1988 Measurement of light within thin plant tissues with fiber optic microprobes. Physiologia Plantarum 72, 623–630.

[CIT0066] Vogelmann TC Martin G 1993 The functional significance of palisade tissue: penetration of directional versus diffuse light. Plant, Cell and Environment 16, 65–72.

[CIT0067] Vogelmann TC Martin G Chen G Buttry D 1991 Fibre optic microprobes and measurement of the light. Advances in Botanical Research 18, 255–295.

[CIT0068] von Caemmerer S 2000 Biochemical models of leaf photosynthesis. Canberra, Australia: CSIRO.

[CIT0069] von Caemmerer S 2013 Steady-state models of photosynthesis. Plant Cell and Environment 36, 1617–1630. 10.1111/pce.12098 23496792

[CIT0070] Wada M 2013 Chloroplast movement. Plant Science 210, 177–182. 23849124 10.1016/j.plantsci.2013.05.016

[CIT0071] Watté R Aernouts B Van Beers R Herremans E Ho QT Verboven P Nicolaï B Saeys W 2015 Modeling the propagation of light in realistic tissue structures with MMC-fpf: a meshed Monte Carlo method with free phase function. Optics Express 23, 17467. 26191756 10.1364/OE.23.017467

[CIT0072] Wise RR Hoober JK 2007 The structure and function of plastids. Dordrecht: Springer Netherlands.

[CIT0073] Wylie RB 1943 The role of the epidermis in foliar organization and its relations to the minor venation. American Journal of Botany 30, 273–280.

[CIT0074] Xiong D Yu T Liu X Li Y Peng S Huang J 2015 Heterogeneity of photosynthesis within leaves is associated with alteration of leaf structural features and leaf N content per leaf area in rice. Functional Plant Biology 42, 687–696. 10.1071/FP15057 32480712

[CIT0075] Yamori W Makino A Shikanai T 2016 A physiological role of cyclic electron transport around photosystem I in sustaining photosynthesis under fluctuating light in rice. Scientific Reports 6, 20147. 26832990 10.1038/srep20147 PMC4735858

[CIT0076] Zhu XG Wang Y Ort DR Long SP 2013 e-photosynthesis: a comprehensive dynamic mechanistic model of C3 photosynthesis: from light capture to sucrose synthesis. Plant Cell and Environment 36, 1711–1727. 10.1111/pce.12025 23072293

[CIT0077] Zurzycki J 1955 Chloroplasts arrangement as a factor in photosynthesis. Acta Societatis Botanicorum Poloniae 24, 27–63.

[CIT0078] Zurzycki J 1961 The influence of chloroplast displacements on the optical properties of leaves. Acta Societatis Botanicorum Poloniae 30, 503–527.

