# Supplementary material for: The influence of leaf anatomy on the internal light environment and photosynthetic electron transport rate: exploration with a new leaf ray tracing model
Source: J Exp Bot. 2016 Oct 4;67(21):6021–35. doi: 10.1093/jxb/erw359 (PMC5100017; doi:10.1093/jxb/erw359)
Supplement: Supplementary Data [file supp_67_21_6021__index.html]

The influence of leaf anatomy on the internal light environment and photosynthetic electron transport rate: exploration with a new leaf ray tracing model — Supplementary Data 

# The influence of leaf anatomy on the internal light environment and photosynthetic electron transport rate: exploration with a new leaf ray tracing model

## Supplementary Data

Data files

- supplementary\_table\_S1.xlsx - Supplementary Data
- supplementary\_figures\_S1\_S6.pdf - Supplementary Data
