# Supplementary material for: The influence of leaf anatomy on the internal light environment and photosynthetic electron transport rate: exploration with a new leaf ray tracing model
Source: J Exp Bot. 2016 Oct 4;67(21):6021–35. doi: 10.1093/jxb/erw359 (PMC5100017; doi:10.1093/jxb/erw359)
Supplement: Supplementary Data [file supp_erw359_supplementary_figures_S1_S6.pdf]

The Influence of Leaf Anatomy on the Internal Light Environment and Photosynthetic Electron Transport Rate

■ Exploration with a new leaf ray tracing model

Yi Xiao, Danny Tholen, Xin-Guang Zhu

Supplementary figures

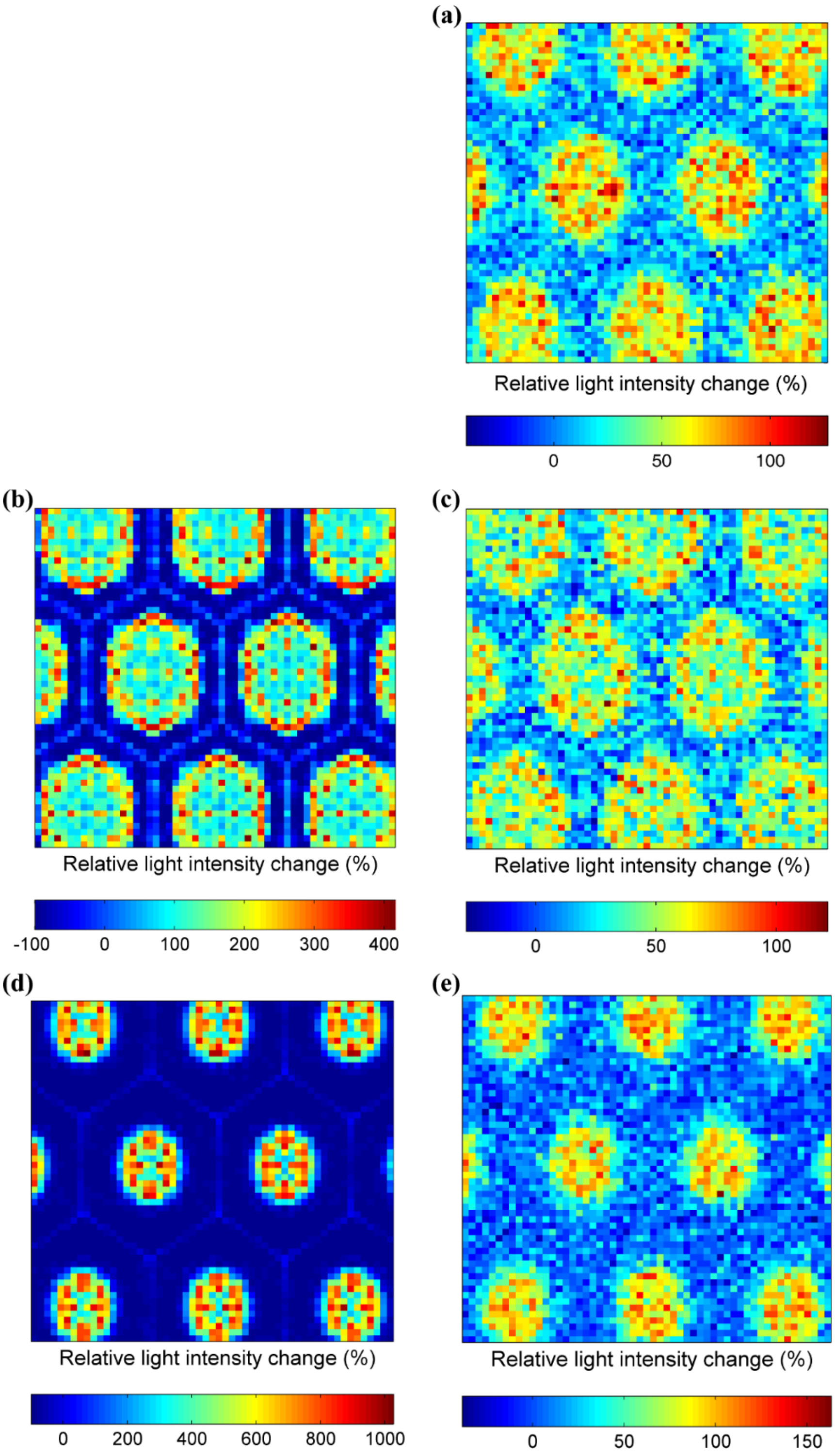

Figure S1. Distribution of light after transmission through epidermal cells with an oblateness of 0.3 **(a)**, 0.5 **(b, c)** and 0.1 **(d, e)** under direct incident light **(b, d)** and diffuse incident light **(a, c, e)**. Different color represents the relative change, which is the percentage change of local incident PFD relative to the incident PFD on the leaf surface.

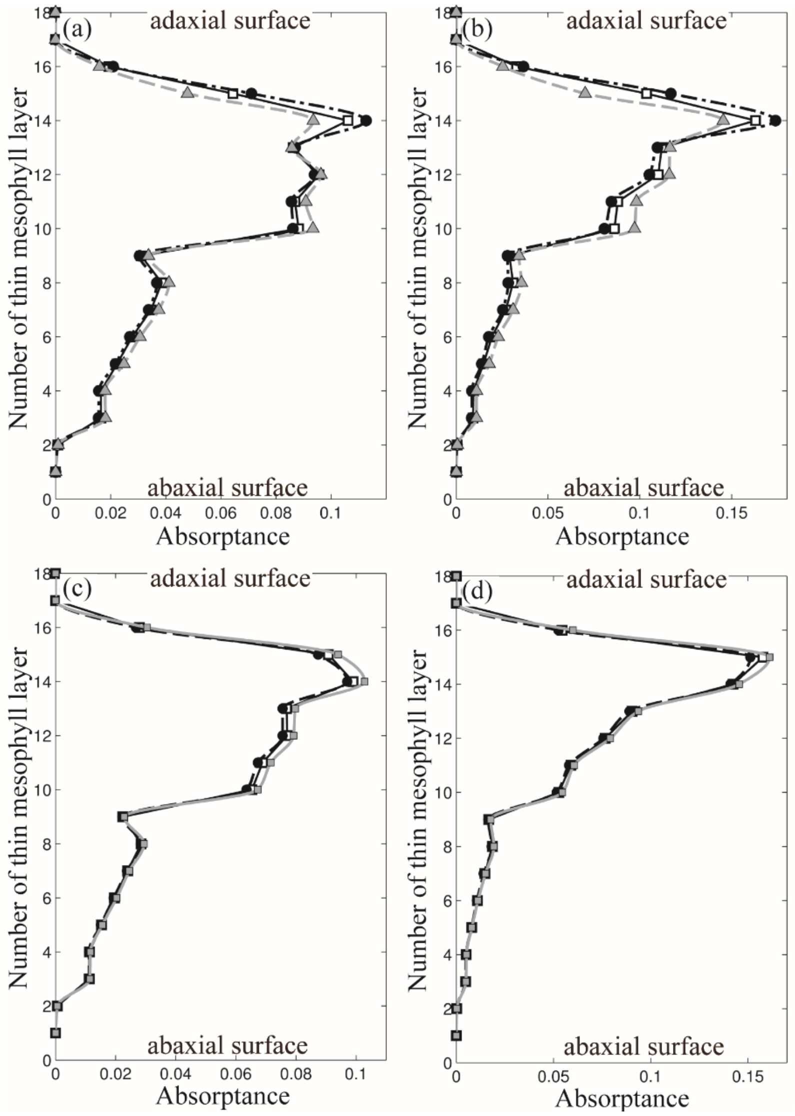

Figure S2. The effect of epidermal oblateness on the distribution of light absorptance across leaf depth under direct green (a) and red (b) light and diffuse green (c) and red (d) light. Each thin mesophyll layer represents 1/18th of the total leaf thickness. Open squares represent results with an epidermis oblateness of 0.3, gray triangles represent results with an oblateness of 0.5 and black circles represent results with an oblateness of 0.1.

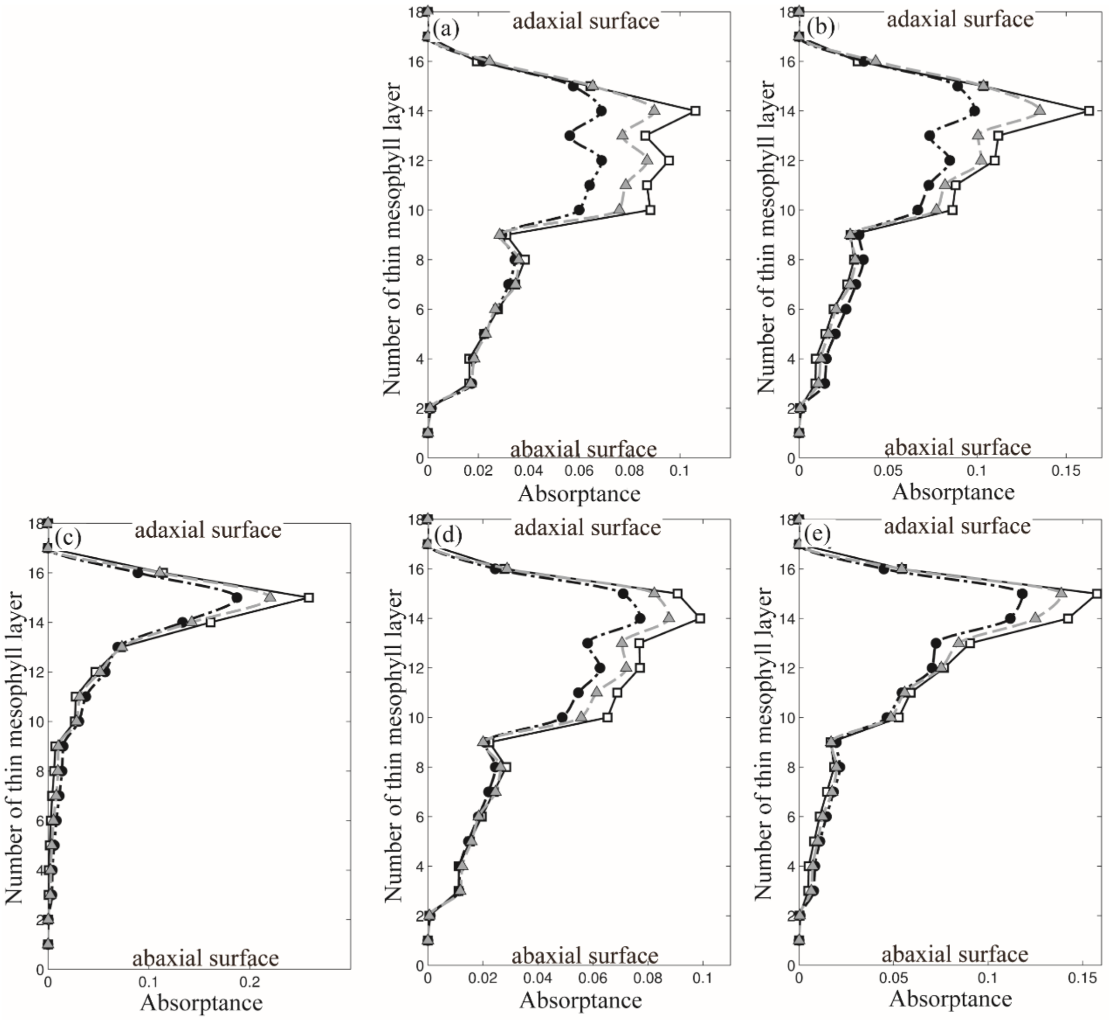

Figure S3. The effect of BSEs on the distribution of light absorptance across leaf depth under direct green (a) and red (b) light and diffuse blue (c), green (d), and red (e) light. Each thin mesophyll layer represents 1/18th of the total leaf thickness. Open squares represented results with no BSEs, gray triangles represented results with 20% BSEs geometry and black circles represented results with 40% BSEs geometry.

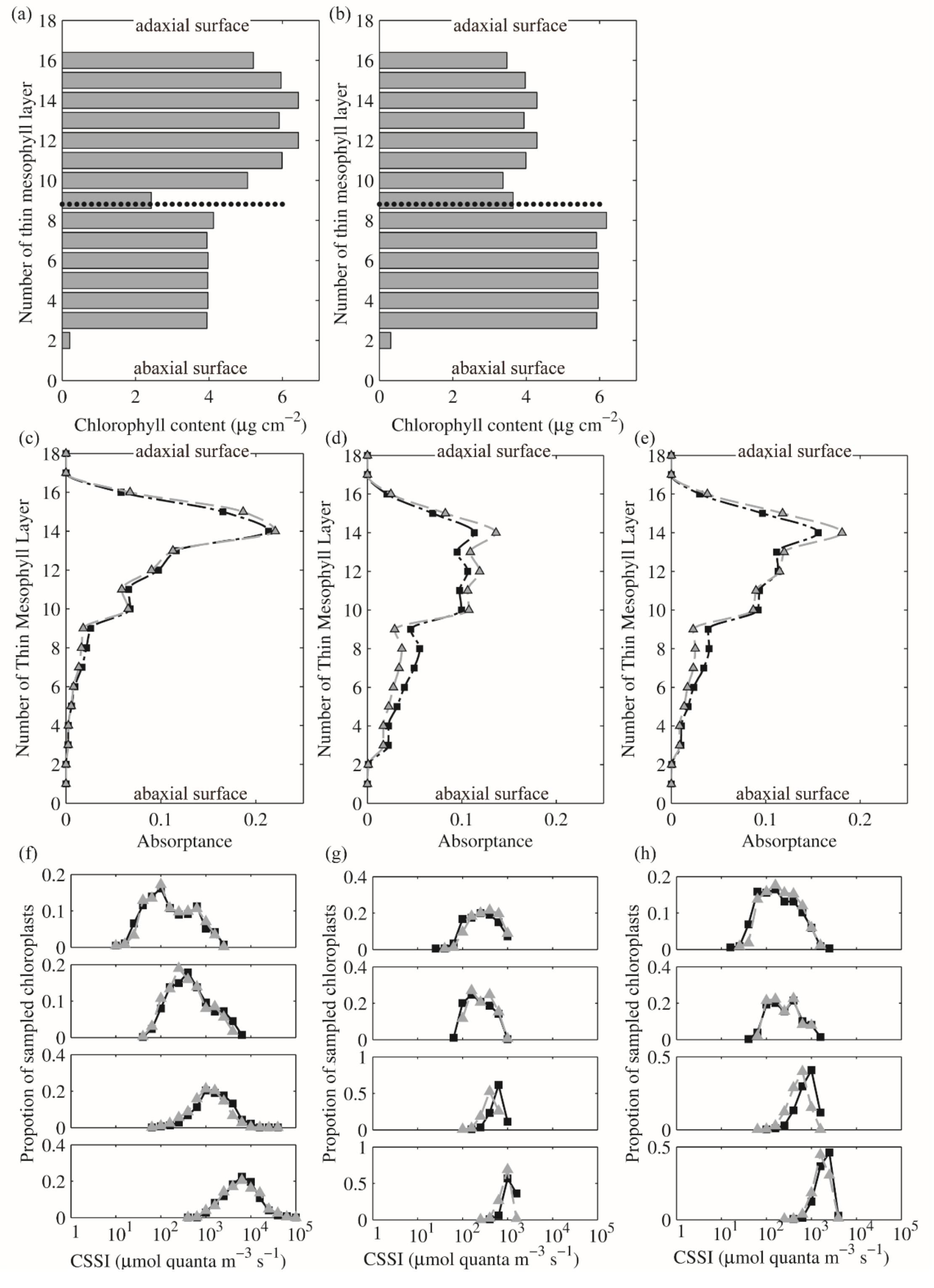

Figure S4. Simulated profile of light absorption under different chlorophyll profiles. Profile of chlorophyll with the chlorophyll concentration in palisade increased or decreased by 20% while the total leaf chlorophyll content was kept the same was shown in **(a)** and **(b)**. And **(c)**, **(d)**, **(e)** showed the predicted light absorption gradient under direct blue, green and red light respectively. Each thin mesophyll layer represents 1/18th of the total leaf thickness. And **(f)**, **(g)**, **(h)** showed the distribution of CSSI in the four previously defined (Figure 2c) sampling regions under direct blue, green and red light. Gray triangles represented results with the chlorophyll profile of (**a**) and black squares represented results with the chlorophyll profile of (**b**).

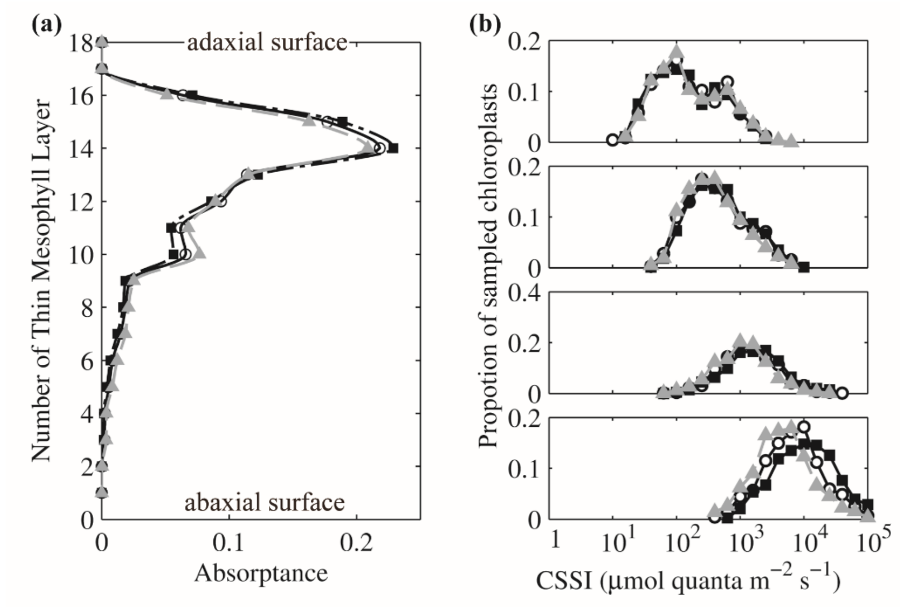

Figure S5. Simulated profile of light absorption under different numbers of chloroplasts. **(a)** The effect of number of chloroplast on the distribution of light absorptance distribution across leaf depth under direct blue light. Each thin mesophyll layer represents 1/18th of the total leaf thickness. **(b)** The distribution of CSSI in the four previously defined (Figure 2c) sampling regions under direct blue light. Open circles represented results with the geometry in Figure 1 of which the chloroplast coverage was around 45%, gray triangles represented results with the chloroplast coverage around 35% and black squares represented results with the chloroplast coverage around 55%.

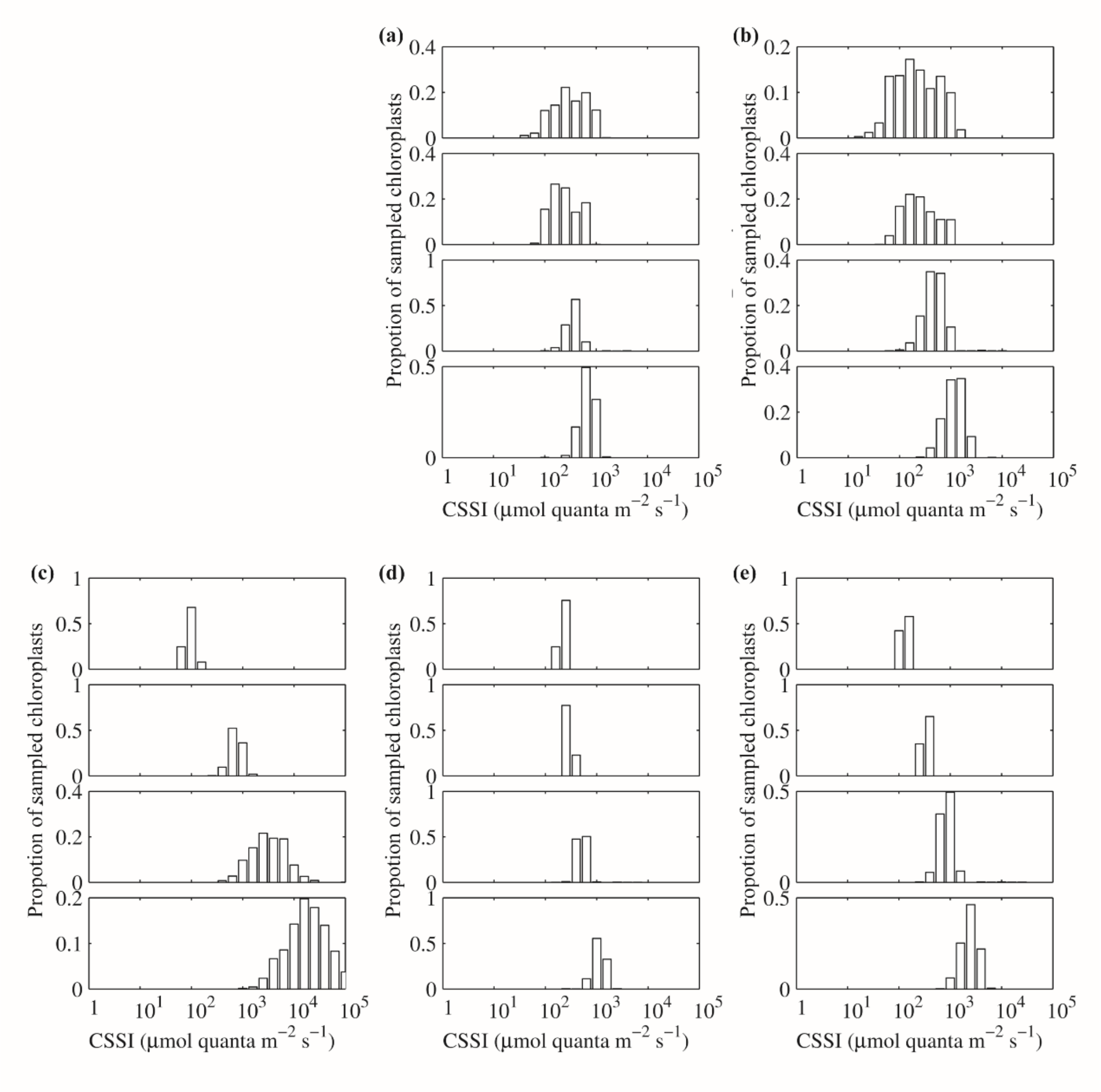

Figure S6. The distribution of CSSI in the four previously defined (Figure 2c) sampling regions under direct green **(a)**, direct red light **(b)**, diffuse blue **(c)**, diffuse green **(d)** and diffuse red **(e)** light.
